# Heavy Metals and Pesticides Toxicity in Agricultural Soil and Plants: Ecological Risks and Human Health Implications

**DOI:** 10.3390/toxics9030042

**Published:** 2021-02-25

**Authors:** Ahmed Alengebawy, Sara Taha Abdelkhalek, Sundas Rana Qureshi, Man-Qun Wang

**Affiliations:** 1 College of Engineering, Huazhong Agricultural University, Wuhan 430070, China; ahmed.alengebawy@yahoo.com; 2 Hubei Insect Resources Utilization and Sustainable Pest Management Key Laboratory, College of Plant Science and Technology, Huazhong Agricultural University, Wuhan 430070, China; staha2015@yahoo.com (S.T.A.); sundasqureshi.pk@hotmail.com (S.R.Q.); 3 Department of Entomology, Faculty of Science, Ain Shams University, Cairo 11566, Egypt

**Keywords:** heavy metal pollution, pesticide risks, toxic effect, agroecosystem, health implications

## Abstract

Environmental problems have always received immense attention from scientists. Toxicants pollution is a critical environmental concern that has posed serious threats to human health and agricultural production. Heavy metals and pesticides are top of the list of environmental toxicants endangering nature. This review focuses on the toxic effect of heavy metals (cadmium (Cd), lead (Pb), copper (Cu), and zinc (Zn)) and pesticides (insecticides, herbicides, and fungicides) adversely influencing the agricultural ecosystem (plant and soil) and human health. Furthermore, heavy metals accumulation and pesticide residues in soils and plants have been discussed in detail. In addition, the characteristics of contaminated soil and plant physiological parameters have been reviewed. Moreover, human diseases caused by exposure to heavy metals and pesticides were also reported. The bioaccumulation, mechanism of action, and transmission pathways of both heavy metals and pesticides are emphasized. In addition, the bioavailability in soil and plant uptake of these contaminants has also been considered. Meanwhile, the synergistic and antagonistic interactions between heavy metals and pesticides and their combined toxic effects have been discussed. Previous relevant studies are included to cover all aspects of this review. The information in this review provides deep insights into the understanding of environmental toxicants and their hazardous effects.

## 1. Introduction

Due to enormous economic development and rapid growth in many fields, such as agriculture and industry, the environment is becoming more polluted [1]. Environmental pollutants are toxic substances that enter the environment from both anthropogenic and natural sources. Certain environmental processes, such as synthetic industries, coal conversion, and waste burning, result in hazardous problems for abiotic elements (water, air, and soil) and biotic communities (animals, plants, and humans) [2]. Usually, environmental toxicants include heavy metals and pesticides, and threaten the entire ecosystem, seriously damaging its function and structure [3].

Naturally, heavy metals are metals with a high atomic weight and a density greater than 5 g/cm^3^ [4]. Compared with their physical properties, the chemical characteristics of heavy metals are the most practical aspects. Environmental toxicity exceeding standard maximum residue limits (MRL) has received heightened consideration from think tanks worldwide [5,6]. Cadmium (Cd), lead (Pb), copper (Cu), and zinc (Zn) cause an alarming combination of environmental and health problems [4,5]. Heavy metals arise from many sources, such as industry, mining, and agriculture. In terms of the sources in the agricultural sector, these can be categorized into fertilization, pesticides, livestock manure, and wastewater [7]. Recently, the risk of heavy metals pollution in the environment has been increasing rapidly and creating turmoil, especially in the agricultural sector, by accumulating in the soil and in plant uptake [6]. The heavy metals contamination problem has become urgent, and needs radical and practical solutions to reduce the hazards as much as possible.

Even though heavy metals are needed for several organs of both plants and humans, they become toxic when their concentration exceeds the prescribed level. Many studies have been done in this area, reporting that the primary sources of heavy metals are agriculture, mining, agrochemicals, and industry. A study done by Xiao et al. [8] reported that agriculture and industry significantly influence heavy metals pollution in agricultural soil and plants, especially soils near cement and electroplating factories. In other words, the soil surface is a fertile place for storing heavy metals, and then transferring them to the plants by absorption along with water through the roots followed by the vascular system.

Heavy metal accumulation can be described as an aggregation of elements in the ecosystem. Plant roots are the essential point of contact for heavy metal ions transmitted from the soil. They tend to stabilize and connect the pollutants in the soil, therefore reducing their bioavailability. The mechanisms of heavy metals transmission to plants include (i) phytoextraction (subprocess of phytoremediation in which plants eliminate hazardous components from contaminated soil), (ii) phytostabilization (immobilization and reduction of the mobility of heavy metals in soil), and (iii) rhizofiltration (a form of phytoremediation to use plant roots to absorb the toxic substances). These metals cause damage to plants, and extend to harm human health through transference in the food chain [9,10].

Recently, due to the rapid evolution of technology, the ecosystem and humans have been exposed to many types of chemical toxicants, in particular, pesticides (herbicides, insecticides, and fungicides) [11,12]. Scientists have defined pesticides as synthesized chemical compounds used in many areas, including in the agricultural sector, to control pests [13]. Therefore, pesticides are considered as efficient, economical, and effective weapons in integrated pest management systems (IPMs) [14]. The uncontrolled use of pesticides causes their bioaccumulation in food chains, which leads to high risk to mammals and other non-target organisms [15]. In addition, the direct or indirect effect of pesticides on non-target organisms leads to an imbalance of the surrounding ecosystem [16]. Moreover, the pesticide residues remain in the plant parts, soil, air, and even penetrate into water [17]. Such residues are considered as one of the most destructive threats the ecosystem faces; these can exist in the environment for a long time, with carcinogenic effects [12].

The deleterious health problems caused by toxicants are increasing due to their penetration and accumulation through the food chain, and their persistence in the ecosystem [18]. Such contaminants can cause acute and chronic diseases in the human body, such as lung cancer, renal dysfunction, osteoporosis, and cardiac failure [19]. Tong et al. [20] mentioned the degree of human health threats posed by heavy metals in China’s urban areas during the period 2003–2009. The results showed that human health risk reports for heavy metals suggested that absorption was the primary route of exposure that has harmful effects on human health. The accumulation of heavy metals in internal human tissues can affect the central nervous system, and act as a pseudo-co-factor or promotor of some health problems, such as seizures (epilepsy), headache, and coma. Heavy metal contamination is considered as a health threat to both adults and children [21]. Pesticides are also hazardous to humans and other living organisms through contaminated food, water, or inhalation of contaminated air [22]. Exposure (whatever the level) to pesticides is hazardous to the behavior and physiology of humans [23]. Furthermore, pesticides are linked with a wide range of diseases, such as hypersensitivity, cancer, asthma, and hormonal disturbances. In addition, they can also lead to congenital disabilities, reduce birth weight, and even cause death [24,25].

Overall, in light of the aforementioned ecological risks, this review covers the sources of heavy metals, the classification of pesticides, and the types of both toxicants. It also discusses the properties of agricultural soils that change due to the contamination of heavy metals and pesticides. In addition, the harmful effects on different plant species ranging from infection to death have been discussed. The novelty of this study is to provide an integrated synthesis of knowledge on the complete pathway of both heavy metals and pesticides starts from their various sources, accumulation in soil and plant, and then reaching human beings. In addition, the synergistic and antagonistic interactions between heavy metals and pesticides, and their combined toxicity in soil, plants, and humans have been reported.

## 2. Sources of Heavy Metals

Scientists divided the sources of heavy metals into two major groups; natural and anthropogenic sources. Natural sources, such as sedimentary rocks, volcanic eruptions, soil formation, and rock weathering, while anthropogenic sources include industry, agriculture, mining, and domestic effluents [26]. However, pollution indicators are an effective mechanism for characterizing soil anthropogenic and geogenic pollution. Nevertheless, it should be noted that source apportionment may be difficult in many cases, despite sophisticated research technics applied [27]. Alloway [28] discussed the different sources of heavy metals and their origin variation, which include sedimentation of aerosol particles, raindrops containing heavy metal, and agrochemicals. Although the study reported many types of metals, it mainly focused on Cd, Pb, Cu, and Zn, which are the same studied heavy metals in this study.

### 2.1. Natural Sources of Heavy Metals

Among the natural sources of heavy metals, igneous and sedimentary rocks are considered as the most common. The concentration ranges (ppm) of heavy metals in the igneous and sedimentary rocks are listed in Table 1. It has been found that elements that existed in one rock type have varying proportions, as well as proportions of different elements vary from one rock type to another [29]. Heavy metals concentration can be determined according to the type of rocks and the surrounding ecosystem conditions [30]. In addition, soil formation is also considered as one of the main reasons of heavy metals accumulation besides river sediments.

### 2.2. Anthropogenic Sources of Heavy Metals

**Figure 1:**
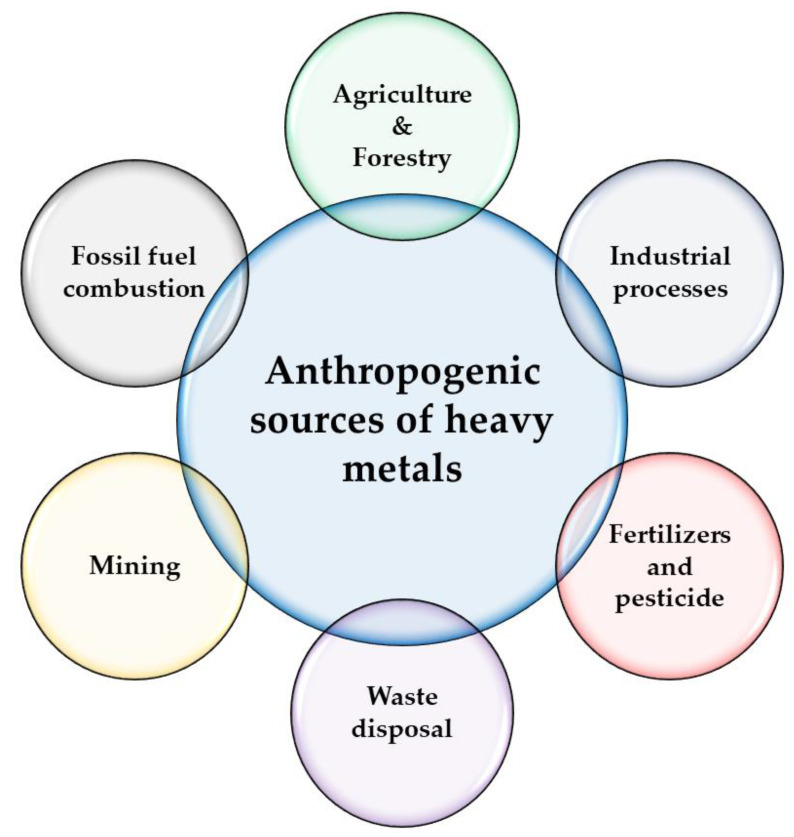
Anthropogenic sources of heavy metal pollution.

Industries, agriculture, mining, and wastewater are deemed anthropogenic sources of heavy metals. These sources significantly lead to the elevation of heavy metals concentration and pollution in the ecosystem, e.g., smelting that results in releasing Cu, Zn, and As; insecticides that contribute to release As; burning of fossil fuels that produces Hg, and cars exhaust that assists in releasing Pb [32]. In addition, daily human activities, such as farming, industrial processes, and manufacturing, impair the balance of the biosphere [33]. Figure 1 shows preeminent anthropogenic sources of heavy metals.

### 2.3. Agricultural Sources of Heavy Metals

Agroecosystems are usually affected by many types of pollutants, including agricultural pollutants, which are known as biotic and abiotic byproducts of farming practices. These pollutants usually cause contamination and degradation of the surrounding agroecosystem. Among the agricultural sources of heavy metals, fertilizers, pesticides, and sewage sludge are the most common [28]. The toxic heavy metals vary in nature and the way of accumulation, whether in soil or plants.

#### Fertilizers as a Source of Heavy Metals Accumulation in Agricultural Soil and Plant

Fertilizers supply different necessary nutrients to enhance plant growth and productivity and increase the soil organic matter. Thus, fertilizers improve soil fertility [34]. Fertilizers can be classified into organic (natural) and inorganic (synthetic) fertilizers. Organic or biofertilizers are produced after the anaerobic digestion (AD) process in the form of ammonium fertilizers (sulfate and nitrate) [35]. Inorganic fertilizers, also known as chemically manufactured/synthetic fertilizers, are a mix of inorganic substances and chemical materials [36]. Fertilizers, including organic and inorganic elements, are responsible for producing heavy metals in the soil. According to the region, Table 2 presents a comparison between the worldwide and European Union (EU), and how heavy metals in different types of fertilizers are varying. Phosphorus is widely used in fertilizer synthesis; simultaneously, it plays a significant role in heavy metals accumulation through its application to the soil [37]. Water-insoluble phosphorus fertilizers have been shown to produce phosphate rocks, which play a major role in the immobilization of metals by precipitation as metal phosphates in the soil [38]. Excessive use of fertilizers for a long time resulting in heavy metals accumulation in agricultural soils reduces soil fertility, and consequently decreases plant growth and productivity [39]. It is extremely challenging to recover the soil environment after heavy metals contaminate the soil. Cu, Zn, and Cd have a higher accumulation potential in agricultural soil due to the long-term use of fertilizers [40]. Phosphate fertilizers, liming materials, and bio-fertilizers are the main types of inorganic fertilizers that contribute to the release of heavy metals in agricultural soil and are then taken up by plants [41]. Therefore, they enter into the food chain and reach animals and humans [42].

## 3. Pesticides as an Environmental Pollutant

By 2050, the world population is estimated to exceed 10 billion, so the food production needs will spike, and world nations will try to overcome that [43,44]. According to Saravi and Shokrzadeh [45], world populations grow by an estimated rate of about 97 million/year.

**Figure 2:**
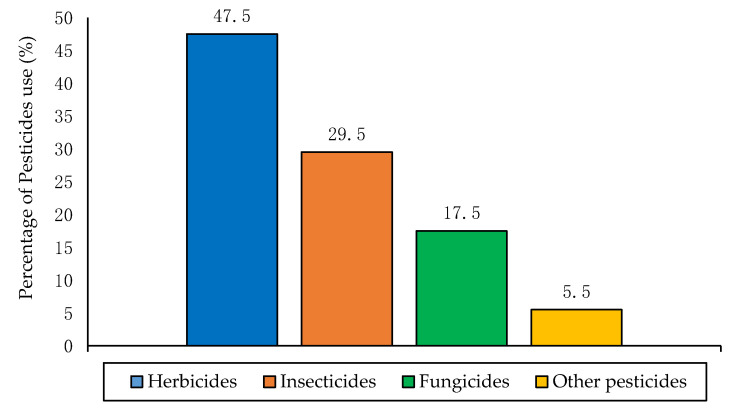
Percentages of global pesticide use (Modified from De et al. [49], Sharma et al. [50]).

Globally, crop injury is caused by approximately 50,000 plant pathogens species, 9000 insects and mites’ species, and 8000 weeds species [46]. These crop injuries are estimated as plant pathogens loss 13%, insect pests loss 14%, and weeds loss 13% [47]. In contrast, pesticides are necessary for plant production, especially the economically important crop species. According to the predictive studies, pesticides protect around one-third of total agricultural products worldwide [48]. Recently, about 2 million tons of pesticides are used globally as 47.5% for herbicides, 29.5% for insecticides, 17.5% for fungicides, and 5.5% for other pesticides [49,50], as shown in Figure 2.

**Figure 3:**
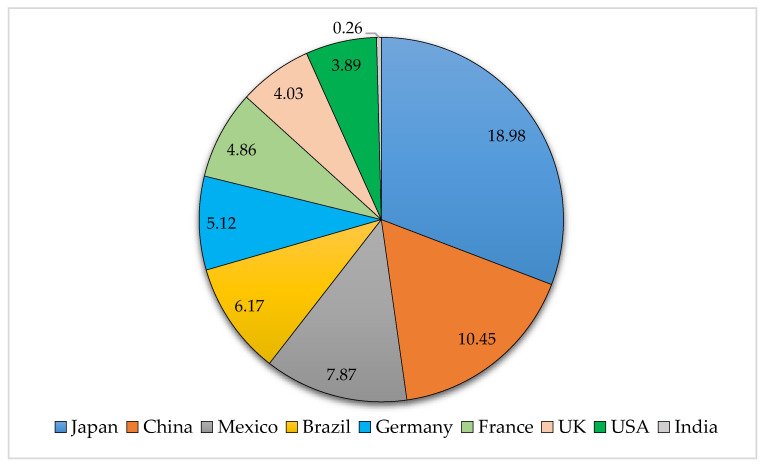
Total averaged pesticide use per year (Kg/ha) during 2010–2014 (Obtained from Zhang [46]).

Globally, the top-ranked pesticide consuming countries are China, followed by the USA, Argentina, Thailand, Brazil, Italy, France, Canada, Japan, and India [51]. Furthermore, Zhang [46] estimated that annual pesticide use would rise to 3.5 million tons globally in 2020. Moreover, he also estimated the total average of the annual pesticide usage (kg/ha) worldwide during 2010–2014, as presented in Figure 3. Japan was the highest with (18.94), while China (10.45), followed by Mexico (7.87), Brazil (6.166), Germany (5.123), France (4.859), UK (4.034), USA (3.886), and finally, India with the smallest value (0.26).

Pesticides are toxic substances or a mixture of substances that are naturally or chemically synthesized. These are widely used for controlling harmful weeds (herbicides), fungi (fungicides), bacteria (bactericides), and insect infestations (insecticides) in the agricultural field [52]. Moreover, these can be used against some disease carriers and pests (e.g., ticks, rodents, mosquitoes, and lice) in the entire ecosystem [22]. Agricultural fields are the largest consumer, which represent about 85% of the global production of pesticides. Furthermore, these can help suppress and prevent insect infestation outbreaks, fungi, and bacteria in moisturized areas (carpets, refrigerators, and cupboards, etc.) [53].

## 4. Pesticides Classification

### 4.1. Major Classes of Pesticides

Pesticides are classified into different groups based on the need of the applicant/user. There are three main classifications as follows: (1) Classification according to pesticides chemical structure, (2) according to the pest they kill, and (3) according to the mode of entry [54,55].

- Classification according to the chemical structure of pesticides

**Figure 4:**
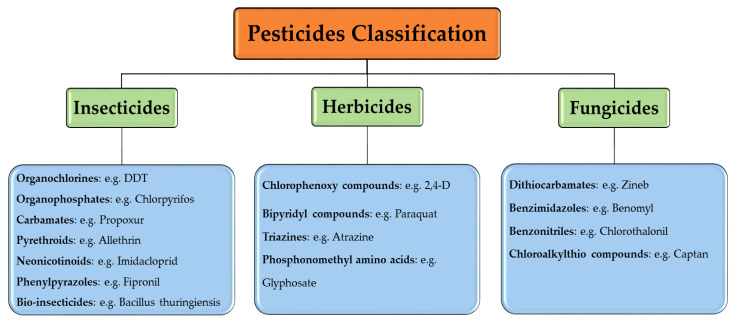
Pesticide classification by pest they kill and the chemical composition (Modified after Yadav and Devi [57]; Fishel and Ferrell [58]).

The most common and helpful style is the classification according to their chemical structure and the nature of active ingredients. This classification is based on the physical and chemical characteristics of pesticide. Such information is beneficial for determining the application method, rate, and the precautions that need to be considered during the application. Within each class, there are many subclasses divided according to their toxicological and chemical composition characteristics. (i) Insecticides contain organochlorines, organophosphorus (OPs), carbamates, pyrethroids, neonicotinoids, and other chemical subclasses; (ii) herbicides contain sulfanilic and carbamic acid, phenylpyrazole, pyridinium, isoxazolyl urea, benzothiazolyl urea, etc.; (iii) fungicides contain carbamates, dithiocarbamates, carboxamides, etc. [55,56]. This subclasses classification is presented in Figure 4.

- Classification according to the pest they kill

This classification category is based on the target pest’s species. The pesticide names reflect their activity by adding the Latin word “cide” (meaning killer) as a suffix after the corresponding name of pest that they kill. For example, insecticides (insect killer), herbicides (weed killer), fungicides (fungi killer), and bactericides (bacteria killer), etc. [56]. Essentially, not all pesticide names contain the suffix “cide” at the end. There are some pesticides named according to their functions, such as repellents (deters insects or pests away from the host), growth regulators, and attractants (attract pests by using some traps like light traps and sex pheromone traps) [59].

- Classification according to the mode of entry

This classification explains how the pesticides enter their target’s body/system. It includes some modes of entry, for example, contact, systemic, stomach poisons, repellents, and fumigants, and detailed definitions and examples are presented in Table 3.

### 4.2. Minor Classes of Pesticides

The minor classes can be categorized into five classes of pesticides: (1) Classification according to the mode of action; (2) according to sources of origin; (3) according to the range of target pests it kills; (4) according to types of a pesticide formulation; (5) according to the toxicity of pesticides [57,60]. In line with our review scope, we will focus only on the last group of minor classes (according to pesticides’ toxicity).

- Classification according to the toxicity of pesticides

Pesticide’s toxicity is the capability to cause injury to an organism. It is determined by exposing target organisms to a varying dosage of a particular formulation, according to hazardous health effects associated with toxic pesticide behavior. The World Health Organization (WHO) divided them into four types [61]. A lab experiment on rats was conducted by WHO to administrate the pesticide doses (orally and dermally). Then LD_50_ (median lethal dose, which kills 50% of the extensive experimental animal population) was calculated. The four categories ranked from the lowest to highest toxicity and expressed a certain toxicity level are shown in Table 4.

## 5. Effect of Heavy Metals Toxicity on Agricultural Soil and Plants

**Figure 5:**
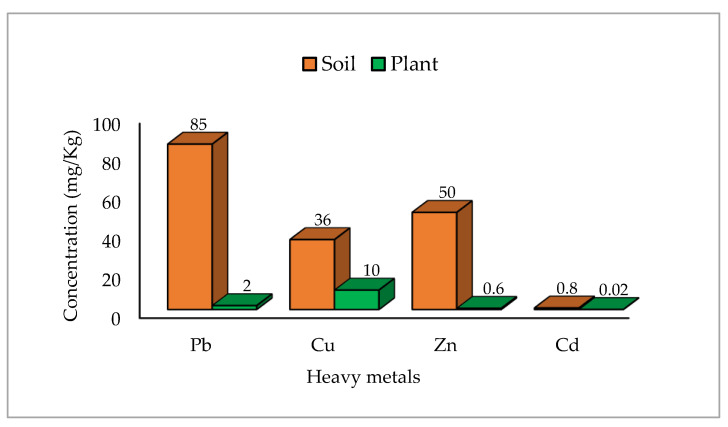
Permissible limits for concentrations of heavy metals in the soil and plants (Modified after WHO [62])**.**

As everyone knows, the high level of heavy metal concentrations influences both soil and plant. WHO has set the permissible limits/MRL of their concentrations (mg·Kg^−1^) in soil and plant (Figure 5) [62]. The metals with high permissible limits are assumed as safe. In soil, the permissible limits of Pb are the highest, followed by Zn and Cu, while the permissible limits of Cd are the lowest. These limits’ values mean that the accumulation of Cd in the soil, even at a lower concentration, is more toxic than Cu, Zn, and Pb. While in the plants, the limit of Cu is the highest, followed by Pb, Zn, and Cd. On the contrary of the soil, Cu has the safest limits, followed by Pb and Zn, while Cd accumulation in plants is the most serious.

### 5.1. Effect of Heavy Metals Toxicity on Agricultural Soil

Heavy metals are considered as a part of the soil; however, they cause severe damage to the soil and plants when they are highly concentrated. Therefore, they are assumed as toxicants [63]. Chrastný et al. [64] studied the distance between the pollution source and the contaminated soils around a mining area near Olkusz town, Upper Silesia, South Poland. The studied soils were agricultural and forest soils. Their results showed that both soils are suffering from the smelting processes, but at various levels. The heavy metals Cd, Pb, and Zn concentrations were found to be 200, 25, and 20 mg·Kg^−1^, respectively. A study done by Raţiu et al. [65] investigated the heavy metals accumulation and their different concentrations around the Tisza River and its tributaries. Their findings stated that the concentration of Cd, Pb, Cu, and Zn in different studied areas were ranged as (1.3–21), (38–3630), (54–4850), (200–770) mg·Kg^−1^, respectively. These results revealed that the concentrations of the studied metals exceeded the permissible limits indicating their toxicity. The low availability of macro-nutrients and soil acidity are among the main problems associated with the accumulative heavy metals toxicity. These problems need to be overcome for the success of the phytostabilization process to remediate the contaminated soils. A comprehensive study was done by Sutkowska et al. [27], who assessed the content of heavy metals and their source apportionment in the Upper Silesia industrial region, Southern Poland. They used different developed pollution indicators to determine the heavy metals content in various soil layers. Their results stated that the concentration of heavy metal was in the following order from highest to lowest: Pb > Cd > Zn in the shallow layers. While in the deep layers, it was Zn > Cd > Pb. The concentration of all metals exceeded the geochemical background levels, indicating the high toxicity of the studied area.

#### 5.1.1. Effect of Cadmium Toxicity on Agricultural Soil

Among the heavy metals, Cd accumulation in the soil is a ubiquitous problem because of the advanced agriculture technology, economic revolution, and the industry’s rapid development. Usually, soil pH and the content of organic matter are the major factors affected by Cd accumulation. With the decreasing of soil pH, Cd bioavailability increased, indicating a defect in soil properties. In 2015, Zeng et al. [66] studied the accumulation of heavy metals in three agricultural areas of Hunan province, China. The study investigated the content of heavy metals in each area. The results stated that Cd and Hg recorded the highest mean values among three tested areas (1.40 and 14.9 mg·Kg^−1^), respectively, which exceeded the Chinese environmental quality standards for soil. In contrast, as a western country example, Chrastný et al. [67] studied the Cd isotope composition in Olkusz. The study was carried out for two meadows and three forest soil profiles influenced by various contamination sources. Their findings indicated that the upper soils in the forest soil profile revealed that Cd isotope compositions were the heaviest, while the lightest were in the deeper soil humus layer. A comprehensive study done by Liao et al. [68] investigated the toxic effect of Cd on paddy soil properties. They used different kinetic and sigmoid dose-response models to determine ecological doses of Cd. Their results stated that Cd caused inhibitory effects on soil microbial activities, microbial growth, and microbial metabolic processes. Raiesi and Sadeghi [69] also studied the interactive consequences of Cd and salinity on soil microbes and enzymatic activities. Their findings reported the synergistic negative effect of Cd and salinity on soil properties. Moreover, their combined effect caused a reduction of microbial respiration and the content of microbial biomass in soil. Oumenskou et al. [70] measured the heavy metals contamination in agricultural soil using the GIS-based approach. Their results revealed that their concentration exceeded the limits set by WHO and FAO. An et al. [71] confirmed that Cd is highly mobile in the soil and consequently resulted in high toxicity that affected the essential microorganisms, inhibited microbial activities, and absorbed the organic matter in the soil, as well as changed the physicochemical characteristics.

#### 5.1.2. Effect of Lead Toxicity on Agricultural Soil

Pb has been listed as a hazardous heavy metal pollutant due to its high toxicity [72]. Long-term exposure to low concentrations of Pb leads to high toxic levels. The main source of Pb contamination in the soil is its geogenic contribution, which reduces the soil microbial activities. The effects of Pb on soil are several, such as reducing soil nutrients, microbial diversity, and soil fertility [73]. Furthermore, earthworms (*Eisenia fetida*) are usually affected by Pb toxicity, which may cause earthworm mortality. Reducing Pb bioavailability in the soil through phytoremediation or phytostabilization strategies is an important issue that should be a focus of concern [74]. Many studies were done on the effect of Pb on agricultural soil in different geographical locations. Vega et al. [75] reported the impact of soil properties on absorption and retention of Pb. The results indicated that soil pH and cation exchange capacity were the important parameters influenced by Pb accumulation. In addition, Kumar et al. [76] stated that a negative correlation between Pb solubility and soil pH was found, indicating that the accumulation of Pb in the soil causes a defect in the plant absorption system from the soil. Pb also affects soil sorption capacity and humic acid content in the soil, as presented by [77]. Khan et al. [78] studied the single and joint effects of Pb and Cd on the soil microbial communities and some enzymatic activities. The findings showed that the microbial communities were extremely affected by the contamination. In addition, the inhibition of enzymes’ activities has also been noted. The combined toxic effects of Pb and Cd were obvious on the number of bacteria and actinomycetes, which were notably reduced. Naturally, some essential soil factors control the mobility and bioavailability of Pb in the soil, such as soil pH, organic matter, ionic exchange capacity, and texture, which are affected by Pb accumulation in the soil.

#### 5.1.3. Effect of Copper Toxicity on Agricultural Soil

Cu is an important micronutrient that is essential and necessary for plants. In addition, it is a significant element in the soil. Cu toxicity is a type of poisoning that causes a defect in any system in which there are above the supra-optimal levels [79]. In agricultural soil, Cu availability is usually affected by several factors, such as soil pH, since its availability is usually higher in acidic soil than in alkaline and organic matter [80]. The high accumulation rate of Cu in the soil is often due to the use of Cu-based fungicides or because of other agricultural activities. Naturally, the range of Cu concentration in agricultural soil is between 5 and 30 mg·Kg^−1^, but this level depends on the condition and the soil’s location [81]. As an example of a European country, Caetano et al. [82], studied the Cu toxicity in natural soil in Portugal; the ecotoxicological assessment reported a negative correlation between the Cu concentration and urease activity. Urease is one of the extracellular enzymes that breakdown the organic matter of soil [83]. The results mentioned above were consistent with the study done by Gülser et al. [84]. Numerous studies informed that Cu toxicity could significantly inhibit soil microbial activities. Cu toxicity can also destruct cell membrane and cause protein denaturation in microbes. Wang et al. [85] studied the toxic effect of Cu on soil microorganisms and microbial biomasses. It has been found that the microorganisms extremely affected by the toxicity were in the following order: Bacteria > actinomycetes > fungi. A study done by Frenk et al. [86] demonstrated the negative effect of nanoscale Cu in the form of copper oxide (CuO) on the microbial groups of soil, such as Rhizobiales. Although the applied CuO concentration was only 1%, it caused a significant decrease in oxidation potential and changed the community formation. Shaw et al. [87] studied the long-term effect of Cu on agricultural soil functions. The results endorsed that Cu concentrations, which were over 200 mg·Kg^−1^ averted in agricultural soil due to high toxicity.

#### 5.1.4. Effect of Zinc Toxicity on Agricultural Soil

Zn is an important micronutrient, promoting plant growth hormones and proteins [88]. It has an active role in plants’ metabolic and physiological processes since it is involved in sugar consumption. However, the imperilment of Zn toxicity is exhibited in its adverse effect on the soil microorganisms that contribute to enhancing soil fertility and structure [89]. The Zn toxicity has a notable relationship with the soil enzyme’s active sites, replacing certain cations that are crucial for cell performance [90]. Moreover, Barman et al. [91] stated that Zn deficiency affects the soil characteristics such as pH, the content of organic matter, bicarbonate content, and impedes the role of Mg and Fe in the soil. Pietrzykowski et al. [92] studied the content of Zn in contaminated soil located in southern Poland. Zn concentration was 10638 mg Kg^−1^, which exceeded the permissible limits indicating the great harm of this kind of soil to plants and humans. These findings are consistent with Ciarkowska et al. [93], who also demonstrated the high concentration of Zn in the studied soil, which harms the activities of the soil enzymes. Wyszkowska et al. [94] investigated the negative role of heavy metals toxicity on soil characteristics, microbes, and enzymes in Poland. Their study reported that high soil pH increased the bioavailability of Zn. Excessive concentrations of Zn and other metals disrupt the soil homeostasis. Moreover, inhibition of microbial enzymatic activities was also noted.

### 5.2. Effect of Heavy Metals Toxicity on Plants

**Figure 6:**
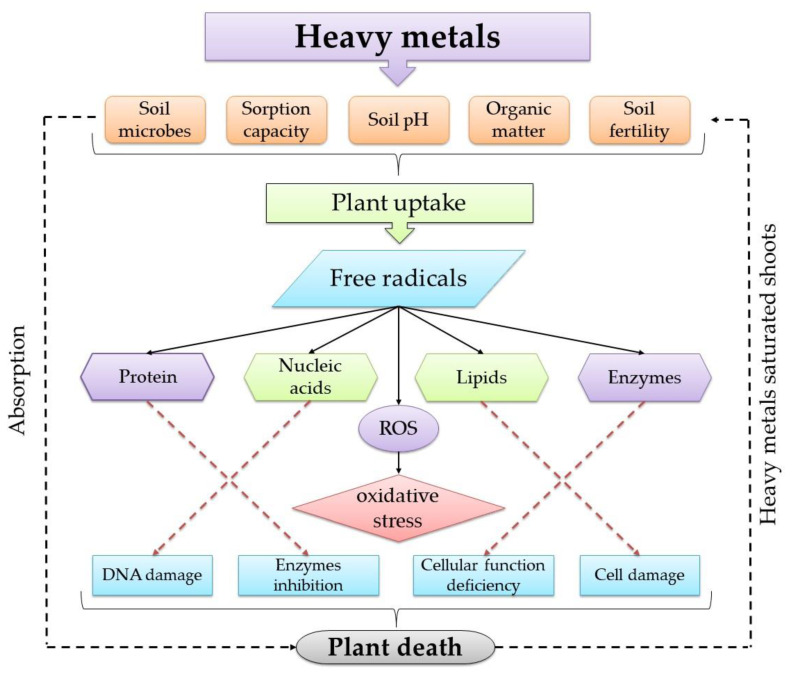
Mechanism of action and pathway of heavy metals toxicity in soil and plant.

Naturally, the plant requires essential elements for growth. Although these trace elements are essential, exposure to heavy metals can severely damage plants. The effect of heavy metals on plants starts in the rhizosphere, where metalliferous minerals and substances interact with root exudates. Cabala and Teper [95] studied the characteristics of the rhizosphere soils polluted by Zn–Pb mining, and thus their negative effect on plant roots. The carbonate formations transpiring on plant roots indicate vital oxidation and dissolution of minerals in the rhizospheres. These processes have been found to increase the metal ion concentrations in solutions of the rhizosphere. Cd toxicity causes a deficiency of minerals in plants [96]. A high concentration of Pb can cause different physiological and biochemical deficiencies [97]. In addition, Cu and Zn interact with each other, affecting the bioavailability of nutrients in the soil. The pathway and mechanism of action of heavy metals, starting from accumulation in the soil passing through the plant uptake reaching different parts of the plant, are shown in Figure 6. The heavy metals produce free radicals, resulting in elevation of intracellular levels of reactive oxygen species (ROS), causing oxidative stress, which causes damage to the biological molecules (e.g., proteins, nucleic acids, lipids, and enzymes). The defect in all these biological molecules causes many physiological problems, such as DNA, cell damage, and the inhibition of enzymatic activities, which may ultimately lead to the death of the entire plant [98].

#### 5.2.1. Effect of Cadmium Toxicity on Plant

Cd is considered as a non-essential and dangerous heavy metal, as it is found in the environment through some anthropogenic sources causing risks to the whole ecosystem [99,100]. According to mobility, bioavailability, and concentration, most Cd ions are absorbed by plant roots; however, the remaining amount can be absorbed directly from the atmosphere [101]. In addition, Cd enters plant cells via other transporters such as Calcium (Ca) channels and accumulate in roots, shoots, and edible parts [100]. A high concentration of Cd in plants causes many physiological and biochemical changes [102]. Furthermore, Cd accumulation in plants results in toxic effects such as inhibition of some processes (minerals transportation, photosynthetic apparatus, and nutrient uptake). In addition, it can inhibit the transportation of Fe into plant shoots [99,103]. Cd has a toxic influence on plant phenotype (e.g., reducing plant weight and length of roots and shoots), cytotoxicity (e.g., reducing chlorophyll content and inhibiting photosynthetic performance), and metabolic processes (e.g., chlorosis and cell damage) [104]. Seregin et al. [105] reported that xylem parenchyma was responsible for heavy metals translocation into conducting tissues; however, the transportation of Cd through the xylem was limited. Gratão et al. [106] stated that Cd toxicity could decrease the roots’ dry mass and length. Recently, a study done by Vardhan et al. [107] investigated the ecological effect of Cd ions on *Lactuca sativa* seeds using *Pichia* sp. and *Azotobacter chroococcum*. The results revealed that Cd (II) inhibited seed germination, reduced the biomass, and hindered the growth and development of roots. In addition, the Cd (II) had a negative impact on microbial growth.

#### 5.2.2. Effect of Lead Toxicity on Plant

Like Cd and mercury (Hg), Pb is not essential for plant growth [108]. Pb is considered as a useful and toxic metal at the same time [109]. It has been categorized as a major pollutant due to its high toxicity [110]. Usually, Pb ions are transported from the soil to the plant via roots through the xylem [111]. Pb toxicity is serious to plants even at low concentrations, which obstructs healthy plant growth and reduces crop yield and productivity [112]. The risk of Pb toxicity in plants is evident in reducing the nutrient uptake and deactivating the permeability of the cell membrane [113]. Pb accumulation in plants causes physiological problems, such as DNA damage and destroying root and shoot systems [114], and affects the enzymatic activities [115]. The effect of Pb toxicity on plants was studied by Nas et al. [116]. The results indicated that a high concentration of Pb affected the fresh biomass and plant growth. The results obtained were consistent with the previous study of Cimrin et al. [117]. A study on soybean crops done by Hamid et al. [118] investigated the toxic effect of Pb on crop growth; the results obtained demonstrated a decrease in the chlorophyll content in the plant. Kushwaha et al. [119] demonstrated that Pb inhibited seed germination and decreased the protein content. They also noted that if the Pb level exceeds the critical threshold, morphological and physiological processes would be severely affected.

#### 5.2.3. Effect of Copper Toxicity on Plant

In terms of worldwide consumption, Cu is ranked third after steel and aluminum. A small amount of Cu is a necessary element for plant nutrition and seed production. However, at high concentrations, Cu is considered a very toxic metal [120,121]. Cu uptake by the plant depends on several factors, such as physicochemical characteristics of the soil and other physiological parameters of the plant [122]. Naturally, the Cu concentration in the roots is higher than in shoots because the root system is responsible for the Cu ions’ uptake from the soil. Kopittke et al. [123] reported that the highest concentration of Cu in the roots was found in the root epidermis. A review article by Adrees et al. [124] stated that Cu toxicity led to a decrease in the crop yield, biosynthesis of chlorophyll, and plant productivity by modification of photosynthesis and nutrients. The concentration of 5 mg Kg^−1^ of Cu is sufficient to harm the plant, reducing plant growth and productivity. For productivity and morphological effects, Barbosa et al. [125] studied the impact of excessive Cu concentration on the maize plant. Their results indicated that plant height was decreased significantly by raising the applied dose of Cu. Aly et al. [126] also studied the negative effects of the high concentration on maize plants. The findings reflected a noticeable decrease in the shoot length, indicating that the toxic effect of Cu reaches all parts of the plant.

#### 5.2.4. Effect of Zinc Toxicity on Plant

Zn is a crucial micronutrient for all living organisms, including plants [127]. Zn is considered as the second most readily available transition metal in organisms after Fe, and it has a strong relation with all enzymatic activities. Usually, Zn is transmitted from the soil as Zn^2+^ and enters the plant via roots [128]. Zn plays a critical role in photosynthetic redox reactions [129]. Accumulation of Zn in plant roots or shoots causes severe damages. Excessive Zn in plant cells causes high turbulence in physiological processes in plants, followed by plant death [130]. A previous study by Ebbs et al. [131] stated that Zn toxicity led to chlorosis for younger leaves at the early stage of exposure, then reached the old leaves. Another study was done by Hammerschmitt et al. [132] on different types of plants to investigate the Zn toxicity on the young peach tree. The results demonstrated that the accumulation of Zn in the root system prevented the elements from transportation to the leaves. Moreover, the dry matter productivity was also negatively affected. Song et al. [127] stated that the exposure to Zn significantly decreased the root length and the photosynthesis. A critical review done by Balafrej et al. [133] summarized the effect of Zn hyperaccumulation in plants and noted that all the physiological and biochemical mechanisms in plants were affected, indicating the harmful effect of Zn accumulation in plants. As a summary of the above-mentioned negative effects of heavy metals, the most common toxic effects of heavy metals (Cd, Pb, Cu, and Zn) on soil and plants are listed in Table 5.

## 6. Effect of Pesticides Toxicity on Agricultural Soil and Plants

The excessive and uncontrolled use of pesticides on different crop species leads to harmful effects on beneficial biota, including honey bees, predators, birds, plants, small mammals, and humans. In addition, these ramifications create an imbalance in the biodiversity of the entire ecological system [14,140,141]. Many systemic pesticides, their derivatives, and metabolites are investigated to be moderately safe to beneficial biota, especially beneficial insects, after their direct contact with such toxicants when they feed on plant tissues. As systemic pesticides can contaminate the floral and extrafloral nectar parts when transmitted systemically through the plant’s vascular system, it leads to high percentages of mortality to honeybees and nectar-feeding parasitoids [142].

**Figure 7:**
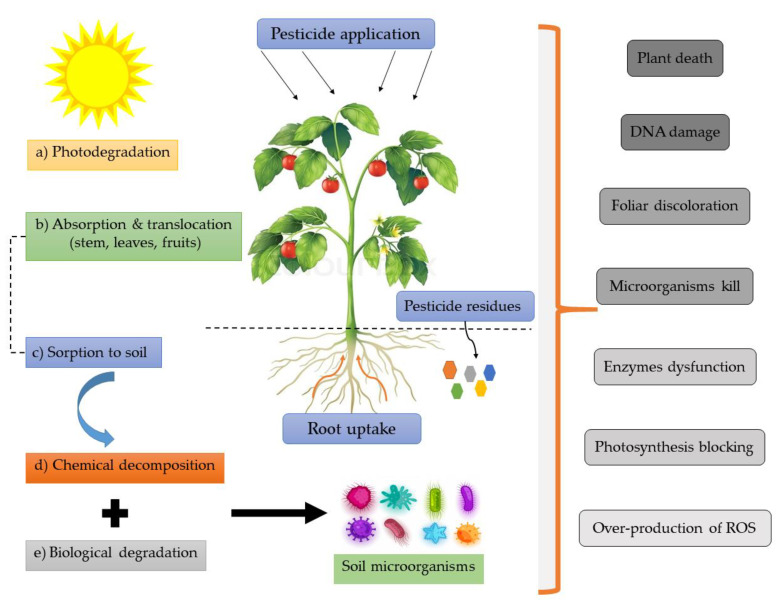
Mechanistic pathway of pesticides toxicity in soil and plant.

Moreover, many pesticides, such as chlordane, dieldrin, hexachlorobenzene thiobencarb, and endrin, resist degradation (persistent organic pollutants) and remain in the environment for a long time. Furthermore, persistent pesticide residues can be bio-accumulated and reach up to a bio-concentration more than 70,000-fold compared with the original concentration [22,143]. A study done by Ligor et al. [144] investigated the residues of five neonicotinoids (thiamethoxam, imidacloprid, acetamiprid, clothianidin, and thiacloprid) that were detected in different honey samples collected from different countries. The concentrations of residues depended on the used amount of pesticides (excessive or moderate use), which reflect the accumulation and the toxic effects of such residues on pollinators (honeybees) and other beneficial organisms. The mechanistic pathway of pesticides starting from the time of application followed by photodegradation, absorption by the plant parts (stem, leaves, or fruit), or sorption at the soil level. Once the pesticides reach the soil, they undergo several biodegradation processes; chemical decomposition (pH, humidity, and temperature) and biological degradation (microorganisms’ enzymes). The pesticides residues and degradation by-products uptake through roots via xylem to the entire plant parts causing some deleterious effects to soil and plant. These effects include overproduction of ROS, oxidative stress, DNA damage, photosynthetic blockage, necrosis, chlorosis, leaves twisting, and ultimately ended with plant death (Figure 7) [145,146].

### 6.1. Effect of Pesticides Toxicity on Agricultural Soil

Many pesticides are being used extensively in the agricultural field to prevent pest damage and improve crop production without considering their harmful effects. As a result of that uncontrolled use, pesticide residues significantly congregate in the soil and increase the contamination, which is directly or indirectly harmful to fauna and flora [147,148]. On the soil level, pesticides can alter the physico-chemical and biological properties of the soil. They can also ultimately disturb microbial activity [149]. Filimon et al. [150] studied the negative impacts of two insecticides (Cypermethrin and Thiomethoxam) on the soil to test a range of physical parameters and determine some biochemical and microbial activities. The results showed that Thiomethoxam leads to a decrease in phosphatase activity by 6.5% compared with control. The number of nitrifying bacteria significantly decreased to 58.1%. Physico-chemical properties were positively correlated with phosphatase, urease, and dehydrogenase activities, while negatively correlated with the aerobic nitrogen-fixing bacteria (*Azothobacter vinellandi*) and nitrifying-bacteria. Moreover, Cypermethrin leads to a decrease in the activity of the dehydrogenase enzyme by 32.8%. Likewise, the number of nitrifying bacteria decreased by 74%. In addition, humidity and pH values were directly proportioned with urease and dehydrogenase activities and the number of nitrifying bacteria as (r > + 0.9) and (r > + 0.8), respectively.

Recently, Al-Ani et al. [151] determined the influence of two insecticides, Miraj (Alphacypermethrin 10%) and Malathion (50% WP), on soil microorganisms (actinomycetes, fungi, and bacteria) and CO_2_ production. The results revealed that CO_2_ production decreased significantly for both insecticides. At week seven, the CO_2_ production values were 32% and 36% for Miraj insecticide concentrations 100 and 200 ppm, respectively. While for Malathion application with 50, 100, and 200 ppm, the CO_2_ production values decreased by 42, 45, and 52%, respectively. Moreover, the number of microorganisms and the microbial activity inversely proportional to the insecticide’s concentrations were added to the soil samples. These results agree with Yousaf et al. [152], who presented the poisonous nature of insecticides to soil microorganisms due to the reduction in the production rate of CO_2_. Goswami et al. [153] showed similar data that applying high concentrations of Cypermethrin insecticide leads to a severe impact on soil respiration and biomass. The effect of organophosphorus insecticides (dimethoate, diazinon, and Malathion) on soil’s microbial communities during 24, 48, and 72 h. The results reflected that microbial growth was significantly inhibited according to the concentration and the exposure time [154]. The repeated application of chlorpyrifos, Malathion, lindane, and endosulfan insecticides reduced the nitrification and denitrification processes in the soil even when these insecticides are applied at the field-recommended doses [155]. Many earlier studies focus on the hazardous effects of different insecticides on various plant species and agricultural soils. Carbamate pesticides inhibit the activity of the nitrogenase enzyme of *Azospirillum* sp. Furthermore, they suppress the growth of various types of soil fauna [156,157]. Niewiadomska [158] stated that imazetapir, carbendazim, and thiram pesticides reduced nitrogenase activity in some plant species, including *Rhizobium trifolii*, *R. leguminosarum*, and *Sinorhizobium melilot* in cultivated samples and under field conditions. Quinalphos decreases soil nitrification and ammonification processes [159,160].

The application of herbicides, especially glyphosate-based herbicides, causes various risks on microbial fauna depending on the application period [161]. Some indirect risks to biodiversity occurred due to the alternation in the physiological and biosynthetic mechanisms of soil ecosystems [162]. Some combinations of herbicides with heavy metals and inorganic fertilizers inhibit the functions of microbial soil communities [163,164]. Such communities are highly intolerant of herbicides’ synergistic interaction with other compounds than the application of a single herbicide [165]. For example, Arif et al. [166] reported that Bromoxynil and Methomyl herbicides decrease the oxidation reaction of methane (CH_4_) to CO_2_. 2,4-D decreases the activity of nitrogenase, phosphatase enzymes, and hydrogen photoproduction of purple non-sulfur bacteria and harm the activities of *Rhizobium* sp. [167,168]. Moreover, Glyphosate leads to a reduction in phosphatase enzyme activity and the growth activity of *azotobacter* [156,169].

Fungicides are classified as the third broadly used pesticide group after insecticides and herbicides that are being used effectively nowadays for crop protection [170]. Furthermore, they cause harmful effects on non-target organisms such as soil microbial communities and influence soil biochemical processes like respiration [171,172]. Baćmaga et al. [173] investigated that the urease enzyme was the most sensitive to the excessive exposure of azoxystrobin. Baćmaga et al. [174] reported that Falcon 460 EC fungicide significantly suppresses the activity of alkaline phosphatase, acid phosphatase, catalase, urease, and dehydrogenase enzymes in soil samples. In addition, overexposure to these active ingredients causes harmful effects on soil-dwelling microbial communities. Fungicides can neutralize soil enzyme activity due to the alleviation of some substances, such as compost and manure [175,176]. Saha et al. [177] reported that tebuconazole has short-term hazardous effects on soil enzymatic activities (arylsulfatase, phosphatase, urease, and fluorescein). Baćmaga et al. [176] stated that the Chlorothalonil affected the soil microbial communities and the biochemical properties. Moreover, they lead to the stimulation of heterotrophic and actinobacteria. The hazardous effects of Chlorothalonil were observed with applications higher than the recommended doses.

### 6.2. Effect of Pesticides Toxicity on Plants

Plant transpiration facilitates the absorption of pesticides, which are soluble in soil, into all parts of the plant [50]. Pesticides translocation occurs through the root system, followed by the vascular system. The presence of their metabolites in the plant vascular system is determined by factors like their reactions with soil and plant, doses of applied pesticide, biochemical and physicochemical properties of pesticide, and the mechanism of pesticide entry [60]. The deleterious effects of pesticides on plants can be detected as chlorosis, burns, leaves twisting, stunting, and necrosis [146,178]. The exposure to organophosphorus insecticide chlorpyrifos suppressed the nitrogen metabolism and growth of *Vigna radiata* L. (mung bean) [179]. Parween et al. [180] investigated chlorpyrifos insecticide’s metabolism and the response of the anti-oxidative enzymatic system of *Vigna radiata* L. during the different stages of growth after the application. The results showed that chlorpyrifos increased the rate of lipid peroxidation and proline content at a concentration of 1.5 mM during the post-flowering time. Moreover, ascorbate and glutathione levels significantly declined during all developmental stages. The activity of antioxidant enzymes increased with all concentrations during the pre-flowering stage. Sharma et al. [178,181] found that Imidacloprid insecticide caused a reduction in the levels of many phytochemical substances in *Brassica juncea*, mustard plant.

Spraying herbicides around the vegetative parts of plants negatively affects the flowering and seed production of plants [182]. Such changes cause pigment discoloration and affect the antioxidant enzymes involved in the defense system, lipid peroxidation, and endogenous hormone levels of non-target plants [183]. Kaya and Doganlar [184] reported that the application of imazapic herbicide induces some phytotoxic effects for tobacco plants, including carotenoids, jasmonic acid, antioxidant enzyme activity, and malondialdehyde contents. Recently, Fernandes et al. [185] investigated the effect of glyphosate-based herbicides (GBH) on a non-target plant (*Medicago sativa* L.). The results reflected an increase in lipid peroxidation that leads to the suppression of roots and shoots growth. Overall, GBH-contaminated soils can greatly destroy the development of non-target plants.

The excessive use of fungicides exerts risky influences on plants (during different growth stages) that cannot be eliminated directly [186,187]. The Falcon 460 EC fungicide showed adverse effects on root elongation and seed germination of some plant species, *Sorgo Saccharum*, *Lepidium sativum*, and *Sinapsis alba*. In addition, the most inhibitory effects were observed on *S. alba* [174]. Hydrogen peroxide levels were increased significantly in tomato plants when exposed to different concentrations of Chlorothalonil fungicide [188]. Furthermore, Xia et al. [189] reported negative effects on stomatal conductance, photosynthetic rate, and cellular CO_2_ of the photosynthetic system in cucumber plants. Ijaz et al. [190] examined the activity of five fungicides involved in triazole and strobilurin classes on two growth stages (course of pod development and green floral bud stages). The findings indicated that there was a significant influence on the leaf area. Carbendazim has the most negative bearing on seed germination and a reduction in the pea’s net growth (*Pisum sativum* L.). Three tested fungicides (Carbendazim, kitazin, and hexaconazole) caused considerable consequences like cellular damages, cytotoxicity, and retardation in root morphology. Due to the fungicides stress, photosynthetic pigment formation and grains production was prohibited. Moreover, some morphological disturbances and alternations in the stomatal behavior of pea leaves [191]. As a summary of the above-mentioned hazardous effects of pesticide, the most common toxic effects of pesticides (insecticides, herbicides, and fungicides) on soil and plant are listed in Table 6.

## 7. Synergism and Antagonism between Heavy Metals and Pesticides in Agricultural Soil and Plant

Despite the high toxicity that may occur from a single component, there is another complex interaction, which may occur between two or more toxic mixtures, causing unpredictable toxicity. This co-occurrence between toxicants may increase (synergism) or decrease (antagonism) the joint toxicity, whether in soil or plants. This joint toxicity could be stronger (synergistic), similar (additive), or weaker (antagonistic) than the single one. Some factors are controlling the co-occurrence, such as bioavailability and biotransformation of these toxicants [198].

Up to now, there has been a lack of available data about the joint toxic effects of heavy metals and other chemical compounds, such as pesticides, on agricultural soil and plant. However, some few studies have discussed the combined toxicity of heavy metals and some pesticides, indicating their severe joint toxicity. One of the most common and sensitive soil toxicity indicators is the earthworm [199]. Wang et al. [200] studied the effect of combined toxicity of heavy metal (Cd) with five types of insecticides on the earthworms. Their findings reported that 21 ternary mixtures showed different interactive effects. Among them, 11 mixtures exhibited synergistic effects, while 5 recorded antagonistic effects. These results revealed that synergistic interactions could occur more than antagonistic ones.

There are no adequate studies that reported the synergistic/antagonistic interaction between heavy metals and pesticides in plants as well. Chen et al. [201] reported the synergistic effect of 2,4-dichlorophenol (2,4-DCP) with Zn and Cu in ryegrass-planted soil. The results revealed an increase in the solubility and toxic activity of heavy metals to plant compared with 2,4-DCP free samples. Numerous studies reported the combined interactions and effects of heavy metals and pesticides, and are presented in Table 7.

## 8. Effect of Heavy Metals and Pesticides Toxicity on Human Health

### 8.1. Effect of Heavy Metals Toxicity on Human Health

**Figure 8:**
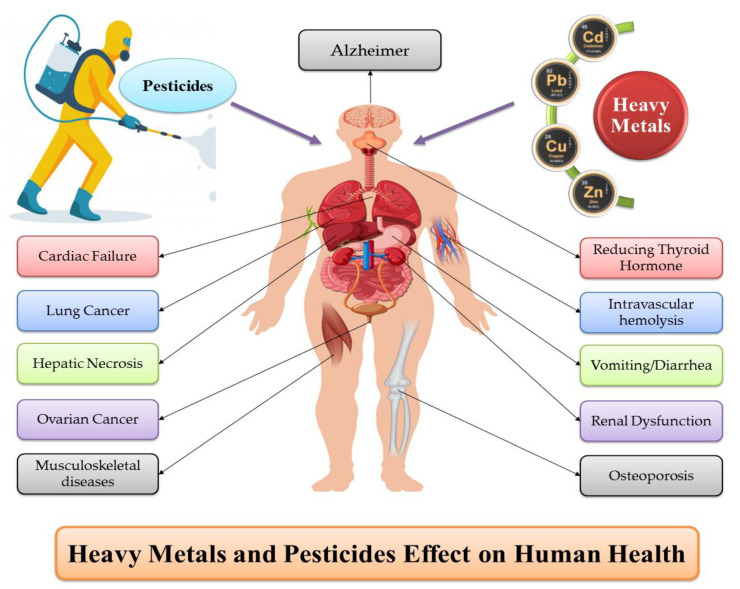
Negative effects of heavy metals and pesticides toxicity on human health.

Metallic elements are naturally existing environmental components found in the earth’s crust, and their compositions vary according to the different origins and regions [211]. Their presence is considered unique because their complete removal from the environment is difficult once they enter it [212]. Due to the toxic effects, long-term accumulation, and bio-magnification characteristics, heavy metal pollution, even at low concentrations, has attracted widespread attention. Heavy metals are considered one of the most critical toxicants among the multilayered soil and environmental pollutants [213,214]. The existence of heavy metals in the ecosystem increases the potential intake of such toxic components by the living organisms and their accumulation in many body organs, including kidney, liver, bone, etc. Moreover, the accumulation of these metals causes deleterious damage to various body systems, such as nervous, skeletal, endocrine, immune, circulatory, etc. [215,216]. Various diseases associated with the toxicity of heavy metals are presented in Figure 8.

Globally, humans are exposed to heavy metals through inhalation (breathing) or ingestion (drinking or eating). People working at or around factories that use these metals and their compounds are at a high risk, as is being near a site where these metals have been illegally discharged. Lifestyle subsistence can also face higher risks of exposure and health effects related to hunting and fishing practices. Recently, the effect of these toxic substances on human health is an intense concern due to ubiquity of exposure. According to the increased use of a wide variety of metals in manufacturing and our daily life through modern applications, problems resulting from the environmental toxic metal emissions have assumed serious dimensions [217].

#### 8.1.1. Effect of Heavy Metal Toxicity on Children’s Health

Toxic heavy metals have certain penetrating mechanisms, including swallowing, dermal absorption, and inhalation, which cause health effects resulting from heavy metals exposure. The effects of heavy metals on children’s health have become more severe than adults. More consideration should be given to heavy metals due to their high toxicity risk, extensive application, and prevalence [218].

According to Chunhabundit [219], Cd toxicity can cause renal damage due to damaged proximal convoluted tubules, which are associated with mitochondrial dysfunction. Moreover, Cd exposure resulted in osteoporosis [220], pediatric cancer, and it has been related to stunted development in children [221]. Gardner et al. [222] stated that Cd exposure was adversely correlated with infant size at birth (height and weight).

Pb exposure is one of the most common preventable poisonings of childhood. Children are particularly vulnerable to Pb toxicity and suffer irreversible neurological deficits affecting the learning ability and behavior [223]. Surma is a popular eye cosmetic paste used as an eyeliner for children in Afghanistan and other countries in the Middle East, Asia, and Africa. It has been confirmed to contain Pb and potentially cause Pb toxicity in infants, leading to permanent damage to multiple organ systems [224]. Evens et al. [225] reported that children exposed to Pb showed inattentiveness, hyperactivity, and irritability. In addition, extremely high Pb exposure levels have been found to cause an increase in dullness, irritability, and shorter attention span in the central nervous system, subsequently resulting in seizures, epilepsy, coma, headache, and even death [226].

Cu is essential to brain function, but it can be toxic if the cellular concentration exceeds the metabolic requirement [227]. In children, elevated serum Cu levels have been associated with impaired working memory. Zhou et al. [228] have proposed that high Cu concentrations can affect working memory through impaired attention. Wilson Disease (WD) is a hereditary disorder predominantly attributed to hepatocellular Cu disposition due to Wilson ATPase dysfunction, a P_1B_-ATPase encoded by the gene ATP7B. Although hepatic disorder is normal in children/adolescents, psychiatric, neurological, and hematological clinical manifestations occur. Very young children may have the clinically evident hepatic disease due to WD [229].

Zn toxicity has been infrequently reported in children. Extreme acute intake has been associated with poor appetite, diarrhea, nausea, headaches, and vomiting. In 2003, Arsenault and Brown [230] found that preschool children had Zn intake that surpassed the dietary reference intake, and about 36% of the children had diets containing Zn exceeded the established upper intake level. In the USA, combining enhanced accessibility of Zn-fortified food coupled with increased usage of Zn supplements could contribute to excessive consumption between children [231]. Zn-induced Cu deficiency was identified due to Cu rivalry at the same absorption site [232]. High oral Zn consumption stimulates metallothionein that binds oral Cu and excretes it out of the body [233]. Moreover, Zn supplements can influence the metabolism of lipoproteins, including low-density lipoprotein, high-density lipoprotein, and cholesterol [234]. Excessive Zn supplementation had been shown to contribute to an impaired immune response [235] and may interfere with numerous medicines, including antibiotics (tetracycline and quinolone) and diuretics such as thiazides [236].

#### 8.1.2. Effect of Heavy Metal Toxicity on Adults

Almost all cells and tissues of the human system could be affected by certain heavy metals. Cd and its compounds can interfere in calcium metabolism, renal tubular dysfunction, osteoporosis, bone diseases, and lung cancer [237]. Neurodegenerative diseases, diabetes, and breast and prostate cancers have been reported to be associated with Cd toxicity [238]. Epidemiological studies documented that exposure to Cd may promote the development of musculoskeletal diseases, such as rheumatoid arthritis, osteoporosis, and osteoarthritis [239]. Cd exposure affects male reproductive systems and semen quality, impairs spermatogenesis, particularly hormonal synthesis/release and sperm motility. According to clinical and human trials, Cd also impairs fertility, reproductive hormonal equilibrium, and affects menstrual cycles [240]. Scientists suspect that Cd may pose a threat to pregnant women. One study suggests that Cd may damage the placenta and reduce the weight of the newborn baby [241]. Cd inhalation can lead to significant damage to the lungs and may even cause death [242].

Pb has a broad spectrum of negative impacts on body systems. Symptoms are predominantly non-specific, for example, decreasing the cognitive function in adults, miscarriage in females, infertility in males, behavioral defects in children. Anemia, renal dysfunction, hypertension, and abdominal colic are also common symptoms [243]. Moreover, Pb is extremely serious to the fetus due to its cross through the placenta, and it can also induce adverse birth effects, including preterm birth [244]. It may cause a reduction of circulating maternal thyroid hormone that impacts overall growth trajectories. Pb results in neurotoxicity, nephrotoxicity disorders and affects heme synthesis [245]. Pb has been reported to give rise to toxicity by substituting Zn for heme synthesis and depleting the function of heme synthesizing enzymes. Various types of neurological syndrome, including Pb encephalopathy and palsy, have been documented to be extremely intoxicated by Pb [246]. It is carcinogenic, and high exposure levels may cause death [247].

Cu is a toxic element found in high concentrations in the brain, liver, and kidneys [248]. Cu toxicity typically induces gastrointestinal (GI) side effects such as stomach pain, hematemesis, melena, jaundice, anorexia, and vomiting combined with erosive gastropathy [249]. In addition, altered mentation, coma, headache, and tachycardia may also accompany GI side effects [250]. Patients with intravascular Cu toxicity (i.e., impaired hemodialysis fluid infusion) may show signs/symptoms of intravascular hemolysis, and individuals with glucose-6-phosphate deficits are at high risk for hematological adverse effects of Cu. Neurological symptoms, such as exhaustion, depression, irritability, agitation, and concentration difficulties were also reported. In most acute forms, Cu toxicity results in rhabdomyolysis, heart and renal failure, methemoglobinemia, intravascular hemolysis, hepatic necrosis, encephalopathy, and eventually mortality [251]. Excess Cu concentrations induce oxidative stress and DNA damage, and reduce cell proliferation [252].

Zn is known to be relatively non-toxic, especially if taken orally. However, manifestations of overt toxicity symptoms (epigastric pain, vomiting, nausea, and fatigue) will occur with elevated intakes of Zn. Although clear symptoms of toxicity require the ingestion of comparatively large quantities of Zn, there is evidence that using Zn supplements by humans can have adverse effects under certain circumstances [253]. Zn-induced neurotoxicity has been shown to play a role in neuronal damage and mortality associated with traumatic brain injury, stroke, epilepsy, and neurodegenerative diseases. During the regular firing of “zincergic” neurons, vesicular free Zn is released into the synaptic cleft, where multiple post-synaptic neuronal receptors are modulated. However, excessive Zn released after injury or illness contributes to excitotoxic neuronal death [254]. Increased Zn levels and oxidative stress are the two main factors in developing amyloid plaques in the neuronal tissue of Alzheimer’s patients [255]. Heavy metal toxicity may result in metabolic syndrome, which describes the co-occurrence of triggers that raise one’s risk of heart disease and other disorders such as diabetes [256].

### 8.2. Effect of Pesticides Toxicity on Human Health

Since the modes of action for pesticides are not species-specific, concerns have been raised about the environmental threats associated with their exposure across different routes (e.g., contaminants in drinking and water food) [22]. Pesticide poisoning is a global public health concern, with almost 300,000 deaths every year worldwide. Pesticide exposure is inevitable; there are multiple methods in which people are exposed to pesticides [257]. Workers in the pesticides sector, transporters of these hazardous substances, farmers, crop vendors, and customers are subjected to various pesticide concentrations [258]. Usually, pesticides are transmitted across the human body by circulation; however, they may be excreted through exhaling air, skin, and urine [14]. There are four common ways pesticides could enter the human body: oral [14], dermal [259], eye [260], and respiratory tract [261]. Pesticide toxicity varies based on the type of exposure, which includes oral, dermal, or respiratory [24].

The risk of pesticide contamination related to health hazards depends not only on how harmful the products are, but also on the extent of the exposure dose [22]. Pesticides toxicity is commonly known to cause only life-threatening diseases such as many types of cancer; however, many other diseases are linked with these toxicants. Most of these disorders can be fatal if they are untreated and compromise an individual’s life quality. Studies also revealed a close relationship between pesticides and cancer development in both adults and children. The people closely associated with pesticide exposure were reported to be at a high-risk level for numerous malignancies, such as neuroblastoma, leukemia, soft tissue sarcoma, Burkitt lymphoma, non-Hodgkin lymphoma, Wilm’s tumor, lung cancer, ovarian cancer, and rectum [262,263,264]. Several epidemiological and clinical studies have documented a relationship between pesticide toxicity and symptoms of bronchial hyper-reactivity and asthma. Pesticide exposure may lead to the exacerbation of asthma by inflammation, irritation, or immunosuppression (Figure 8) [261,265]. Emerging scientific pieces of evidence showed that exposure to environmental toxicants may cause diabetes. Exposure to pesticides, specifically organochlorines and their metabolites, is reported to impart a high risk of developing type 2 diabetes and its comorbidities [266].

Epidemiological studies investigated that environmental factors (e.g., pesticides) play a key role in Parkinson’s disease (PD) initiation. Most studies were conducted to discover a correlation between pesticide exposure and PD. The findings showed a significant positive relationship between them. A potential risk of PD was found to be associated with rotating crops. Furthermore, paraquat was confirmed to be positively correlated with the increased risk of PD. Freire and Koifman [267] and Brouwer et al. [268] documented a relationship between PD and some pesticides use, such as herbicides (paraquat), insecticides (organophosphate and rotenone), and fungicides (cyprodinil, fenhexamid, and thiophanate-methyl). Exposure to such pesticides at adequate doses could increase sperm abnormalities, fetal growth retardation, decrease aberrant fertility abortions, and birth defects risks [269]. The majority of pesticides, including organophosphorus compounds, influence the male reproductive pathways by reducing sperm activities (e.g., counts, viability, density, and motility), suppression of spermatogenesis, decreasing testis weights, and impairing sperm DNA [270]. Observational research of workers exposed to pesticides, concurrently with laboratory animal models, demonstrated how these compounds are responsible for detrimental effects on health. Nevertheless, understanding the molecular mechanism of how pesticides influence human health is critical. Pesticides inhibit the activity of endocrine hormones, release time, or imitate these hormones, which decrease fertility and cause genital tract abnormalities in both males and females [271]. Simultaneously, they cause altered immune function and numerous forms of cancer [272]. Genetic disruption by pesticides can be generally divided into three main classes; (i) pre-mutagenic damages like DNA strand breaks [273], DNA adducts [274], (ii) gene mutations, such as insertions, deletions, inversions, and translocation [275], (iii) chromosomal aberrations, including loss or gain of the entire chromosome (aneuploidy), deletion or breaks (clastogenicity), and chromosomal rearrangements [276]. Genetic changes due to pesticide exposure resulting in polymorphisms leading to an altered affinity to their ligand or modification in the expression of the downstream target genes [277].

Although pesticides are designed to deter, eliminate, or control undesirable pests, many studies have raised questions about the environmental and human health risks associated with such pesticides. Hence, natural biological control agents (viruses, insects, beneficial bacteria, and nematodes) can be used as control strategies. In addition, all stakeholders, including governmental departments, nongovernmental organizations, and producers should make broad-spectrum efforts in research, quality enhancement, product monitoring and registration, and introduce pesticide usage policies while advocating public education concerning pesticides.

### 8.3. Combined Toxic Effects of Mixtures of Heavy Metals and Pesticides on Human Health

Daily exposure to multiple toxicants increases the threat to human body organs. In a combined form, heavy metals and pesticides pose an upsurge to this menace. Only a little is known about the toxicity of heavy metal-pesticide mixture. Nevertheless, these mixtures may cause impulsive repercussions due to their interaction with each other and with the environment [278]. The literature also lacks knowledge about cellular and molecular changes occurring in human cells and altering human genetics. Some studies conducted on animal models, including but are not limited to a study that found that the spermatogenic element was lost significantly, together with disorganization and seminiferous epithelium and lacking maturation of germs cells in rats due to the combined effect of Cd and diazinon [279]. Cytotoxicity analysis of Cd and chlorpyrifos mixture resulted in lipid accumulation in hepatocytes, causing an increase in hepatic toxicity [280]. A recent investigation on patients related to different occupations with pancreatic cancer exposed to pesticides found that they had higher concentrations of Cd and Mn [281]. Researchers hypothesized that exposure to chemical mixtures occurring at high concentrations and the influence of co-exposure of multiple toxicants need to be further studied.

## 9. Conclusions

This review highlighted the toxic repercussions of heavy metals and pesticides on three important components of the ecosystem (soil, plants, and humans). The harmful effects of heavy metals and pesticides were comprehensively discussed. In addition, their implications on human health were also observed. Cd is extremely mobile in the soil and consequently affects the essential microorganisms and absorbs soil’s organic matter. Soil pH and sorption capacity can be negatively affected by Pb accumulation. Moreover, Cu has a harmful effect on soil microbial groups, such as Rhizobiales. Whereas Zn can inhibit the activities of beneficial microbes and bacteria. Cd causes inhibition of minerals transportation and negatively affects plant’s microbial growth. Pb accumulation in plants causes DNA damage, chlorophyll content reduction, and inhibition of seed germination. Decreasing crop yield and biosynthesis of chlorophyll are the most negative consequences of Cu toxicity. Moreover, Zn blocks the translocation of nutrients to leaves and decreases photosynthesis causing plant death.

The discussion mentioned above highlights that pesticide residues cause direct and indirect damage to fauna, flora, physicochemical, and biological properties of agricultural soil. Furthermore, they can decrease the enzymatic activity and inhibit the soil microbial communities. Pesticides can cause chlorosis, necrosis, leaves twisting, and photosynthesis malfunctioning due to oxidative stress. Furthermore, many studies investigated that different classes of pesticides lead to nitrogen metabolism suppression, increasing and decreasing some enzymes activity. Moreover, leaf pigmentations can be changed, and fruits and grains may stop growing.

Heavy metals and pesticides cause deleterious implications for human health. Different body organs can be affected along with body systems. Heavy metals toxicity causes serious problems for children and adults by ingestion, inhalation, and dermal adsorption. The harmful health implications of heavy metals can be concluded as neurodegenerative disorders, musculoskeletal diseases, and reproductive hormonal imbalance. Pesticide exposure causes hazardous effects, such as soft tissue sarcoma, ovarian cancer, lung cancers, asthma, and endocrine disruption. Moreover, they cause genetic damages. They also play an important role in Parkinson’s disease promotion and the DNA damage of sperm. As future perspectives and recommendations, the co-occurrence of toxic mixtures, their interactions, and combined toxicity must be investigated in detail. We also suggest that further studies should be carried out on new approaches to the phytoremediation and bioremediation of environmental toxicants.

## Figures and Tables

**Table 1:** Range of heavy metal concentrations (ppm) in igneous and sedimentary rocks (Modified from Cannon et al. [31]).

Metals	Basaltic Igneous	Granite Igneous	Shales and Clays	Black Shales	Sandstone
Cd	0.006–0.6	0.003–0.18	0.0–11	<0.3–8.4	-
Pb	30–160	4–30	18–120	20–200	-
Cu	48–240	5–140	18–180	34–1500	2–41
Zn	2–18	6–30	16–50	7–150	<1–31

**Table 2:** Comparison between heavy metal concentrations (mg·Kg^−1^) in different types of fertilizers and livestock manure around the world and in the EU (Modified from Alloway [28]).

Heavy Metals	P Fertilizers		N Fertilizers		Lime Fertilizers		Manure Fertilizers	
	Worldwide	EU	Worldwide	EU	Worldwide	EU	Worldwide	EU
Cd	0.1–170	13	0.05–8.5	0.9	0.04–.01	0.2	0.3–0.8	–
Pb	1.0–300	26	1.0–15	2.0	2.0–125	5.6	2.0–60	–
Cu	7.0–225	13	2–1450	1.9	20–1250	8.2	6.6–350	–
Zn	50–1450	236	1.0–42	5.0	10–450	22	15–250	–

**Table 3:** Pesticides classification according to the mode of entry (Modified from Yadav and Devi [57]).

Mode of Entry	Definition	Example
Contact pesticides	They enter the target’s body by direct contact (especially the physical contact). This type of pesticide enters the body via the epidermal layer.	Diquat dibromide
Systemic pesticides	They are absorbed by the plant vascular system, then translocate to the remaining untreated tissues.	Glyphosate
Stomach poisons	They enter the target’s body via their digestive tract during their food ingestion, followed by death due to the poisoning.	Malathion
Repellents	They do not enter the target’s body and kill them; they only push back and resist the pests to keep them away from the host.	Methyl anthranilate
Fumigants	They kill the pests by producing vapor (gaseous state) of the pesticide. These vapors enter the pest’s body through the spiracles (tracheal system).	1,3-dichloropropene

**Table 4:** Classification according to the toxicity of pesticides set by the World Health Organization (WHO) [61].

WHO Type	Toxicity Level	LD_50_ for the Rat(mg/kg Body Weight)		Examples
		Oral	Dermal	
Type Ia	Extremely hazardous	<5	<50	Parathion, Dieldrin
Type Ib	Highly hazardous	5–50	50–200	Eldrin, Dichlorvos
Type II	Moderately hazardous	50–2000	200–2000	DDT, Chlordane
Type III	Slightly hazardous	>2000	>2000	Malathion

**Table 5:** Heavy metal toxicity forms and their toxic effects on soil and plants.

Heavy Metals	Toxicity Form	Toxic Effects		References
		Soil	Plant	
Cd	Cd^+2^	Kill microorganisms, absorb organic matter, and change soil physicochemical characteristics.	Reduce biomass and root length, inhibit seed germination, and reduce stem conductivity.	[70,99,107,134,135]
Pb	Pb^+2^	Change soil pH, affect soil sorption capacity, and reduce soil fertility.	DNA damage, decrease chlorophyll content, decrease protein content, and cause stunted foliage.	[32,74,135,136]
Cu	Cu salts	Change urease activity, affect microbial communities, and decrease oxidation potential.	Root deformation, decrease shoot length, reduce polypeptides, and change in lipid content.	[82,86,135,137]
Zn	Zn^+2^	Change bicarbonate and organic matter content, inhibit enzymatic activity, and affect soil pH.	Variation in enzymatic activity, obstruction of elements transmission, and cause interveinal chlorosis.	[93,135,138,139]

**Table 6:** Toxic effects of pesticides on agricultural soil and plant.

Pesticide Type	Toxic Effects		References
	Soil	Plant	
Insecticides	Destruction of microbial structural proteins, symbiotic attributes reduction, change soil chemistry and enzymatic activity.	Reduction in grain protein content, blockage of stomatal conductance, and alterations in the photosynthetic process.	[192,193]
Herbicides	Reduction of the soil nutrients availability and suppression of phosphatase and nitrogenase activities.	Alteration of the physiological and biochemical plant efficiency, increasing the susceptibility of plants toward diseases.	[156,194]
Fungicides	Interruption of phosphatase, urease, and dehydrogenase activities and inhibition of the nitrifying bacterial growth.	Reduction of chlorophyll and carotenoid concentrations, destruction of chloroplasts, stomatal closure, and electron transfer suppression.	[195,196,197]

**Table 7:** Interaction between heavy metals and pesticides and their joint toxic effect on agricultural soil and plant.

Heavy Metal	Pesticide	Joint Interaction	Joint Toxic Effect	References
Soil organisms				
Cd	λ-cyhalothrin Chlorpyrifos, Atrazine	Antagonism Synergism	Earthworm mortality.	[200]
Pb	Acetochlor Glyphosate	Synergism Antagonism	Na Change soil pH.	[202,203]
Cu	Acetochlor Cypermethrin	Synergism Synergism & Antagonism	Earthworm mortality. Change catalase activity.	[204,205]
Zn	Chlorpyrifos 2,4-DCP	Synergism Antagonism	Reduce acetylcholinesterase activity. Limited effect on Zn dissolution.	[201,206]
Plant parts				
Cd	Acetochlor Bensulfuron-methyl	Synergism Synergism	Decline soluble protein content, Affect nitrate reductase activity, suppression of roots and shoots growth.	[207]
Pb	Acetochlor	Synergism & Antagonism *	Root elongation inhibition.	[202]
Cu	Glyphosate	Synergism & Antagonism *	Change tissue structure of the cell membrane, severe production of ROS, membrane lipids peroxidation.	[208,209]
Zn	Glyphosate	Antagonism	Reduce the phytotoxicity of destructive weeds.	[210]

Na: not available; * concentration-dependent.

## References

[1] Ali H., Khan E., Ilahi I. (2019). Environmental chemistry and ecotoxicology of hazardous heavy metals: Environmental persistence, toxicity, and bioaccumulation. J. Chem..

[2] Bhunia P. (2017). Environmental Toxicants and Hazardous Contaminants: Recent Advances in Technologies for Sustainable Development. J. Hazard. Toxic. Radioact. Waste.

[3] Chin N.P. (2010). Environmental toxins: Physical, social, and emotional. Breastfeed. Med..

[4] Zhang X., Yan L., Liu J., Zhang Z., Tan C. (2019). Removal of different kinds of heavy metals by novel PPG-nZVI beads and their application in simulated stormwater infiltration facility. Appl. Sci..

[5] Su C., Jiang L., Zhang W. (2014). A review on heavy metal contamination in the soil worldwide: Situation, impact and remediation techniques. Environ. Skept. Critics.

[6] Tóth G., Hermann T., Da Silva M.R., Montanarella L. (2016). Heavy metals in agricultural soils of the European Union with implications for food safety. Environ. Int..

[7] Li Z., Ma Z., van der Kuijp T.J., Yuan Z., Huang L. (2014). A review of soil heavy metal pollution from mines in China: Pollution and health risk assessment. Sci. Total Environ..

[8] Xiao R., Wang S., Li R., Wang J.J., Zhang Z. (2017). Soil heavy metal contamination and health risks associated with artisanal gold mining in Tongguan, Shaanxi, China. Ecotoxicol. Environ. Saf..

[9] Cho-Ruk K., Kurukote J., Supprung P., Vetayasuporn S. (2006). Perennial plants in the phytoremediation of lead-contaminated soils. Biotechnology.

[10] Tangahu B.V., Sheikh Abdullah S.R., Basri H., Idris M., Anuar N., Mukhlisin M. (2011). A Review on Heavy Metals (As, Pb, and Hg) Uptake by Plants through Phytoremediation. Int. J. Chem. Eng..

[11] Pastor S., Creus A., Parrón T., Cebulska-Wasilewska A., Siffel C., Piperakis S., Marcos R. (2003). Biomonitoring of four European populations occupationally exposed to pesticides: Use of micronuclei as biomarkers. Mutagenesis.

[12] Özkara A., Akyıl D., Konuk M., Larramendy M.L., Soloneski S. (2016). Pesticides, environmental pollution, and health. Environmental Health Risk-Hazardous Factors to Living Species.

[13] Cooper J., Dobson H. (2007). The benefits of pesticides to mankind and the environment. Crop Prot..

[14] Damalas C.A., Eleftherohorinos I.G. (2011). Pesticide Exposure, Safety Issues, and Risk Assessment Indicators. Int. J. Environ. Res. Public Health.

[15] Liu Y., Li S., Ni Z., Qu M., Zhong D., Ye C., Tang F. (2016). Pesticides in persimmons, jujubes and soil from China: Residue levels, risk assessment and relationship between fruits and soils. Sci. Total Environ..

[16] Rosell G., Quero C., Coll J., Guerrero A. (2008). Biorational insecticides in pest management. J. Pestic. Sci..

[17] Lefrancq M., Imfeld G., Payraudeau S., Millet M. (2013). Kresoxim methyl deposition, drift and runoff in a vineyard catchment. Sci. Total Environ..

[18] Verger P.J.P., Boobis A.R. (2013). Reevaluate pesticides for food security and safety. Science.

[19] Ying L., Shaogang L., Xiaoyang C. (2016). Assessment of heavy metal pollution and human health risk in urban soils of a coal mining city in East China. Hum. Ecol. Risk Assess. An Int. J..

[20] Tong S., Li H., Wang L., Tudi M., Yang L. (2020). Concentration, Spatial Distribution, Contamination Degree and Human Health Risk Assessment of Heavy Metals in Urban Soils across China between 2003 and 2019—A Systematic Review. Int. J. Environ. Res. Public Health.

[21] Lu X., Zhang X., Li L.Y., Chen H. (2014). Assessment of metals pollution and health risk in dust from nursery schools in Xi’an, China. Environ. Res..

[22] Kim K.H., Kabir E., Jahan S.A. (2017). Exposure to pesticides and the associated human health effects. Sci. Total Environ..

[23] Mascarelli A. (2013). Growing up with pesticides. Science.

[24] Sharon M., Bhawana M., Anita S., Gothecha V.K. (2012). A short review on how pesticides affect human health. Int. J. Ayurvedic Herb. Med..

[25] Wickerham E.L., Lozoff B., Shao J., Kaciroti N., Xia Y., Meeker J.D. (2012). Reduced birth weight in relation to pesticide mixtures detected in cord blood of full-term infants. Environ. Int..

[26] Roozbahani M.M., Sobhanardakani S., Karimi H., Sorooshnia R. (2015). Natural and Anthropogenic Source of Heavy Metals Pollution in the Soil Samples of an Industrial Complex; a Case Study. Iran. J. Toxicol..

[27] Sutkowska K., Teper L., Czech T., Hulok T., Olszak M., Zogala J. (2020). Quality of Peri-Urban Soil Developed from Ore-Bearing Carbonates: Heavy Metal Levels and Source Apportionment Assessed Using Pollution Indices. Minerals.

[28] Alloway B.J., Alloway B.J. (2013). Sources of Heavy Metals and Metalloids in Soils. Heavy Metals in Soils. Trace Metals and Metalloids in Soils and their Bioavailability.

[29] Bradl H.B., Bradl H.B. (2005). Sources and origins of heavy metals. Interface Science and Technology.

[30] Sharma R.K., Agrawal M. (2005). Biological effects of heavy metals: An overview. J. Environ. Biol..

[31] Cannon H.L., Connally G.G., Epstein J.B., Parker J.G., Thornton I., Wixson G. (1978). Rocks: Geological sources of most trace elements. Report to the Workshop at South Scas Plantation Captiva Island, FL, US. Geochem Environ.

[32] Masindi V., Muedi K.L., Saleh H.E.-D.M., Aglan R.F. (2018). Environmental Contamination by Heavy Metals. Heavy Metals.

[33] He Z.L., Yang X.E., Stoffella P.J. (2005). Trace elements in agroecosystems and impacts on the environment. J. Trace Elem. Med. Biol..

[34] Meng L., Alengebawy A., Ai P., Jin K., Chen M., Pan Y. (2020). Techno-Economic Assessment of Three Modes of Large-Scale Crop Residue Utilization Projects in China. Energies.

[35] Alengebawy A., Jin K., Ran Y., Peng J., Zhang X., Ai P. (2021). Advanced pre-treatment of stripped biogas slurry by polyaluminum chloride coagulation and biochar adsorption coupled with ceramic membrane filtration. Chemosphere.

[36] Cai L.M., Wang Q.S., Wen H.H., Luo J., Wang S. (2019). Heavy metals in agricultural soils from a typical township in Guangdong Province, China: Occurrences and spatial distribution. Ecotoxicol. Environ. Saf..

[37] Chen X.X., Liu Y.M., Zhao Q.Y., Cao W.Q., Chen X.P., Zou C.Q. (2020). Health risk assessment associated with heavy metal accumulation in wheat after long-term phosphorus fertilizer application. Environ. Pollut..

[38] Bolan N.S., Adriano D.C., Naidu R., Ware G.W., Albert L.A., Bro-Rasmussen F., Crosby D.G., de Voogt P., Frehse H., Hutzinger O., Mayer F.L., Morgan D.P., Park D.L. (2003). Role of Phosphorus in (Im)mobilization and Bioavailability of Heavy Metals in the Soil-Plant System BT—Reviews of Environmental Contamination and Toxicology: Continuation of Residue Reviews. Reviews of Environmental Contamination and Toxicology.

[39] Ai P., Jin K., Alengebawy A., Elsayed M., Meng L., Chen M., Ran Y. (2020). Effect of application of different biogas fertilizer on eggplant production: Analysis of fertilizer value and risk assessment. Environ. Technol. Innov..

[40] Wang X., Liu W., Li Z., Teng Y., Christie P., Luo Y. (2020). Effects of long-term fertilizer applications on peanut yield and quality and plant and soil heavy metal accumulation. Pedosphere.

[41] Fan Y., Li Y., Li H., Cheng F. (2018). Evaluating heavy metal accumulation and potential risks in soil-plant systems applied with magnesium slag-based fertilizer. Chemosphere.

[42] Liu Y.M., Liu D.Y., Zhang W., Chen X.X., Zhao Q.Y., Chen X.P., Zou C.Q. (2020). Health risk assessment of heavy metals (Zn, Cu, Cd, Pb, As and Cr) in wheat grain receiving repeated Zn fertilizers. Environ. Pollut..

[43] Gill H.K., Garg H. (2014). Pesticide: Environmental impacts and management strategies. Pestic. Asp..

[44] Saravi S.S.S., Dehpour A.R. (2016). Potential role of organochlorine pesticides in the pathogenesis of neurodevelopmental, neurodegenerative, and neurobehavioral disorders: A review. Life Sci..

[45] Saravi S.S.S., Shokrzadeh M., Stoytcheva M. (2011). Role of pesticides in human life in the modern age: A review. Pesticides in the Modern World-Risks and Benefits.

[46] Zhang W. (2018). Global pesticide use: Profile, trend, cost/benefit and more. Proc. Int. Acad. Ecol. Environ. Sci..

[47] Pimentel D., Peshin R., Dhawan A.K. (2009). Pesticides and pest control. Integrated pest management: Innovation-development process.

[48] Zhang W.-J., van der Werf W., Pang Y. (2011). A simulation model for vegetable-insect pest-insect nucleopolyhedrovirus epidemic system. J. Environ. Entomol..

[49] De A., Bose R., Kumar A., Mozumdar S., De A., Bose R., Kumar A., Mozumdar S. (2014). Targeted Delivery of Pesticides Using Biodegradable Polymeric Nanoparticles.

[50] Sharma A., Kumar V., Shahzad B., Tanveer M., Sidhu G.P.S., Handa N., Kohli S.K., Yadav P., Bali A.S., Parihar R.D. (2019). Worldwide pesticide usage and its impacts on ecosystem. SN Appl. Sci..

[51] Top Pesticide Using Countries. https://www.worldatlas.com/articles/top-pesticide-consuming-countries-of-the-world.html.

[52] Abdel Khalek S.T., Mostafa Z.K., Hassan H.A., Abd El-Bar M.M., Abu El-Hassan G.M.M. (2018). A New List to the Entomofauna Associated with Faba Bean, Vicia faba L.(Fabales: Fabaceae) Grown in El-Kharga Oasis, New Valley Governorate, Egypt. Egypt. Acad. J. Biol. Sci..

[53] Gilden R.C., Huffling K., Sattler B. (2010). Pesticides and health risks. J. Obstet. Gynecol. Neonatal Nurs..

[54] Drum C. (1980). Soil Chemistry of Pesticides.

[55] Kaur R., Mavi G.K., Raghav S., Khan I. (2019). Pesticides classification and its impact on environment. Int. J. Curr. Microbiol. Appl. Sci.

[56] Lippmann M., Leikauf G.D. (2020). Introduction and background. Environ. Toxicants Hum. Expo. Their Health Eff..

[57] Yadav I.C., Devi N.L. (2017). Pesticides classification and its impact on human and environment. Environ. Sci. Eng..

[58] Fishel F.M., Ferrell J.A. (2010). Managing pesticide drift. EDIS.

[59] Katagi T., Whitacre D.M. (2010). Bioconcentration, bioaccumulation, and metabolism of pesticides in aquatic organisms. Reviews of Environmental Contamination and Toxicology.

[60] Lushchak V.I., Matviishyn T.M., Husak V.V., Storey J.M., Storey K.B. (2018). Pesticide toxicity: A mechanistic approach. EXCLI J..

[61] (2009). World Health Organization (WHO) Dengue: Guidelines for Diagnosis, Treatment, Prevention and Control: New Edition. https://apps.who.int/iris/handle/10665/44188.

[62] World Health Organization (WHO) (1996). Permissible Limits of Heavy Metals in Soil and Plants.

[63] Osmani M., Bani A., Hoxha B. (2015). Heavy Metals and Ni Phytoextractionin in the Metallurgical Area Soils in Elbasan. Albanian J. Agric. Sci..

[64] Chrastný V., Vaněk A., Teper L., Cabala J., Procházka J., Pechar L., Drahota P., Penížek V., Komárek M., Novák M. (2012). Geochemical position of Pb, Zn and Cd in soils near the Olkusz mine/smelter, South Poland: Effects of land use, type of contamination and distance from pollution source. Environ. Monit. Assess..

[65] Raţiu I.-A., Beldean-Galea M.S., Bocoş-Binţinţan V., Costea D.-D. (2018). Priority Pollutants Present in the Tisza River Hydrographic Basin and their Effects on Living Organisms. Jordan J. Chem..

[66] Zeng F., Wei W., Li M., Huang R., Yang F., Duan Y. (2015). Heavy metal contamination in rice-producing soils of Hunan province, China and potential health risks. Int. J. Environ. Res. Public Health.

[67] Chrastný V., Čadková E., Vaněk A., Teper L., Cabala J., Komárek M. (2015). Cadmium isotope fractionation within the soil profile complicates source identification in relation to Pb-Zn mining and smelting processes. Chem. Geol..

[68] Liao M., Luo Y.K., Zhao X.M., Huang C.Y. (2005). Toxicity of cadmium to soil microbial biomass and its activity: Effect of incubation time on Cd ecological dose in a paddy soil. J. Zhejiang Univ. Sci..

[69] Raiesi F., Sadeghi E. (2019). Interactive effect of salinity and cadmium toxicity on soil microbial properties and enzyme activities. Ecotoxicol. Environ. Saf..

[70] Oumenskou H., El Baghdadi M., Barakat A., Aquit M., Ennaji W., Karroum L.A., Aadraoui M. (2018). Assessment of the heavy metal contamination using GIS-based approach and pollution indices in agricultural soils from Beni Amir irrigated perimeter, Tadla plain, Morocco. Arab. J. Geosci..

[71] An Y.J. (2004). Soil ecotoxicity assessment using cadmium sensitive plants. Environ. Pollut..

[72] Qi X., Xu X., Zhong C., Jiang T., Wei W., Song X. (2018). Removal of Cadmium and Lead from Contaminated Soils Using Sophorolipids from Fermentation Culture of Starmerella bombicola CGMCC 1576 Fermentation. Int. J. Environ. Res. Public Health.

[73] Dotaniya M.L., Dotaniya C.K., Solanki P., Meena V.D., Doutaniya R.K., Gupta D.K., Chatterjee S., Walther C. (2020). Lead Contamination and Its Dynamics in Soil–Plant System. Lead in Plants and the Environment.

[74] Lan M.M., Liu C., Liu S.J., Qiu R.L., Tang Y.T. (2020). Phytostabilization of cd and pb in highly polluted farmland soils using ramie and amendments. Int. J. Environ. Res. Public Health.

[75] Vega F.A., Andrade M.L., Covelo E.F. (2010). Influence of soil properties on the sorption and retention of cadmium, copper and lead, separately and together, by 20 soil horizons: Comparison of linear regression and tree regression analyses. J. Hazard. Mater..

[76] Kumar A., Kumar A., Cabral-Pinto M., Chaturvedi A.K., Shabnam A.A., Subrahmanyam G., Mondal R., Gupta D.K., Malyan S.K., Kumar S.S. (2020). Lead toxicity: Health hazards, influence on food Chain, and sustainable remediation approaches. Int. J. Environ. Res. Public Health.

[77] Placek A., Grobelak A., Kacprzak M. (2016). Improving the phytoremediation of heavy metals contaminated soil by use of sewage sludge. Int. J. Phytoremediation.

[78] Khan S., Hesham A.E.L., Qiao M., Rehman S., He J.Z. (2010). Effects of Cd and Pb on soil microbial community structure and activities. Environ. Sci. Pollut. Res..

[79] Vlcek V., Pohanka M. (2018). Adsorption of copper in soil and its dependence on physical and chemical properties. Acta Univ. Agric. Silvic. Mendelianae Brun..

[80] Keiblinger K.M., Schneider M., Gorfer M., Paumann M., Deltedesco E., Berger H., Jöchlinger L., Mentler A., Zechmeister-Boltenstern S., Soja G. (2018). Assessment of Cu applications in two contrasting soils—effects on soil microbial activity and the fungal community structure. Ecotoxicology.

[81] Brun L.A., Le Corff J., Maillet J. (2003). Effects of elevated soil copper on phenology, growth and reproduction of five ruderal plant species. Environ. Pollut..

[82] Caetano A.L., Marques C.R., Gonçalves F., da Silva E.F., Pereira R. (2016). Copper toxicity in a natural reference soil: Ecotoxicological data for the derivation of preliminary soil screening values. Ecotoxicology.

[83] Cordero I., Snell H., Bardgett R.D. (2019). High throughput method for measuring urease activity in soil. Soil Biol. Biochem..

[84] Gülser F., Erdoǧan E. (2008). The effects of heavy metal pollution on enzyme activities and basal soil respiration of roadside soils. Environ. Monit. Assess..

[85] Wang L., Xia X., Zhang W., Wang J., Zhu L., Wang J., Wei Z., Ahmad Z. (2019). Separate and joint eco-toxicological effects of sulfadimidine and copper on soil microbial biomasses and ammoxidation microorganisms abundances. Chemosphere.

[86] Frenk S., Ben-Moshe T., Dror I., Berkowitz B., Minz D. (2013). Effect of metal oxide nanoparticles on microbial community structure and function in two different soil types. PLoS ONE.

[87] Shaw J.L.A., Ernakovich J.G., Judy J.D., Farrell M., Whatmuff M., Kirby J. (2020). Long-term effects of copper exposure to agricultural soil function and microbial community structure at a controlled and experimental field site. Environ. Pollut..

[88] Njinga R.L., Moyo M.N., Abdulmaliq S.Y. (2013). Analysis of Essential Elements for Plants Growth Using Instrumental Neutron Activation Analysis. Int. J. Agron..

[89] Mertens J., Smolders E., Alloway B.J. (2013). Zinc. Heavy Metals in Soils. Trace Metals and Metalloids in Soils and their Bioavailability.

[90] Łukowski A., Dec D. (2018). Influence of Zn, Cd, and Cu fractions on enzymatic activity of arable soils. Environ. Monit. Assess..

[91] Barman H., Das S.K., Roy A. (2018). Zinc in Soil Environment for Plant Health and Management Strategy. Univers. J. Agric. Res..

[92] Pietrzykowski M., Antonkiewicz J., Gruba P., Pajak M. (2018). Content of Zn, Cd and Pb in purple moor-grass in soils heavily contaminated with heavy metals around a zinc and lead ore tailing landfill. Open Chem..

[93] Ciarkowska K., Gargiulo L., Mele G. (2016). Natural restoration of soils on mine heaps with similar technogenic parent material: A case study of long-term soil evolution in Silesian-Krakow Upland Poland. Geoderma.

[94] Wyszkowska J., Borowik A., Kucharski M., Kucharski J. (2013). Effect of cadmium, copper and zinc on plants, soil microorganisms and soil enzymes. J. Elem..

[95] Cabala J., Teper L. (2007). Metalliferous constituents of rhizosphere soils contaminated by Zn-Pb mining in southern Poland. Water. Air. Soil Pollut..

[96] Nazar R., Iqbal N., Masood A., Khan M.I.R., Syeed S., Khan N.A. (2012). Cadmium Toxicity in Plants and Role of Mineral Nutrients in Its Alleviation. Am. J. Plant Sci..

[97] Shahid M., Pinelli E., Pourrut B., Silvestre J., Dumat C. (2011). Lead-induced genotoxicity to Vicia faba L. roots in relation with metal cell uptake and initial speciation. Ecotoxicol. Environ. Saf..

[98] Wu X., Cobbina S.J., Mao G., Xu H., Zhang Z., Yang L. (2016). A review of toxicity and mechanisms of individual and mixtures of heavy metals in the environment. Environ. Sci. Pollut. Res..

[99] Jibril S.A., Hassan S.A., Ishak C.F., Megat Wahab P.E. (2017). Cadmium Toxicity Affects Phytochemicals and Nutrient Elements Composition of Lettuce ( Lactuca sativa L.). Adv. Agric..

[100] Zhou C., Ge N., Guo J., Zhu L., Ma Z., Cheng S., Wang J. (2019). Enterobacter asburiae Reduces Cadmium Toxicity in Maize Plants by Repressing Iron Uptake-Associated Pathways. J. Agric. Food Chem..

[101] Ismael M.A., Elyamine A.M., Moussa M.G., Cai M., Zhao X., Hu C. (2019). Cadmium in plants: Uptake, toxicity, and its interactions with selenium fertilizers. Metallomics.

[102] Feng J., Shi Q., Wang X., Wei M., Yang F., Xu H. (2010). Silicon supplementation ameliorated the inhibition of photosynthesis and nitrate metabolism by cadmium (Cd) toxicity in Cucumis sativus L. Sci. Hortic. (Amsterdam)..

[103] Schützendübel A., Nikolova P., Rudolf C., Polle A. (2002). Cadmium and H2O2-induced oxidative stress in Populus x canescens roots. Plant Physiol. Biochem..

[104] Hayat M.T., Nauman M., Nazir N., Ali S., Bangash N., Hasanuzzaman M., Prasad M.N.V., Fujita M. (2018). Environmental Hazards of Cadmium: Past, Present, and Future. Cadmium Toxicity and Tolerance in Plants: From Physiology to Remediation.

[105] Seregin I.V., Kozhevnikova A.D. (2008). Roles of root and shoot tissues in transport and accumulation of cadmium, lead, nickel, and strontium. Russ. J. Plant Physiol..

[106] Gratão P.L., Monteiro C.C., Rossi M.L., Martinelli A.P., Peres L.E.P., Medici L.O., Lea P.J., Azevedo R.A. (2009). Differential ultrastructural changes in tomato hormonal mutants exposed to cadmium. Environ. Exp. Bot..

[107] Vardhan K.H., Kumar P.S., Panda R.C. (2019). A review on heavy metal pollution, toxicity and remedial measures: Current trends and future perspectives. J. Mol. Liq..

[108] Diaconu M., Pavel L.V., Hlihor R.M., Rosca M., Fertu D.I., Lenz M., Corvini P.X., Gavrilescu M. (2020). Characterization of heavy metal toxicity in some plants and microorganisms—A preliminary approach for environmental bioremediation. N. Biotechnol..

[109] Uzu G., Sobanska S., Aliouane Y., Pradere P., Dumat C. (2009). Study of lead phytoavailability for atmospheric industrial micronic and sub-micronic particles in relation with lead speciation. Environ. Pollut..

[110] Chauhan P., Rajguru A.B., Dudhe M.Y., Mathur J. (2020). Efficacy of lead (Pb) phytoextraction of five varieties of Helianthus annuus L. from contaminated soil. Environ. Technol. Innov..

[111] Gupta N., Yadav K.K., Kumar V., Kumar S., Chadd R.P., Kumar A. (2019). Trace elements in soil-vegetables interface: Translocation, bioaccumulation, toxicity and amelioration - A review. Sci. Total Environ..

[112] Ashraf U., Kanu A.S., Deng Q., Mo Z., Pan S., Tian H., Tang X. (2017). Lead (Pb) toxicity; physio-biochemical mechanisms, grain yield, quality, and Pb distribution proportions in scented rice. Front. Plant Sci..

[113] Ashraf U., Kanu A.S., Mo Z., Hussain S., Anjum S.A., Khan I., Abbas R.N., Tang X. (2015). Lead toxicity in rice: Effects, mechanisms, and mitigation strategies—a mini review. Environ. Sci. Pollut. Res..

[114] Gichner T., Žnidar I., Száková J. (2008). Evaluation of DNA damage and mutagenicity induced by lead in tobacco plants. Mutat. Res. - Genet. Toxicol. Environ. Mutagen..

[115] Reddy A.M., Kumar S.G., Jyothsnakumari G., Thimmanaik S., Sudhakar C. (2005). Lead induced changes in antioxidant metabolism of horsegram (Macrotyloma uniflorum (Lam.) Verdc.) and bengalgram (Cicer arietinum L.). Chemosphere.

[116] Ali M., Nas F.S. (2018). The effect of lead on plants in terms of growing and biochemical parameters: A review. MOJ Ecol. Environ. Sci..

[117] Cimrin K.M., Turan M., Kapur B. (2007). Effect of elemental sulphur on heavy metals solubility and remediation by plants in calcareous soils. Fresenius Environ. Bull..

[118] Hamid N., Bukhari N., Jawaid F. (2010). Physiological responses of Phaseolus vulgaris to different lead concentrations. Pakistan J. Bot..

[119] Kushwaha A., Hans N., Kumar S., Rani R. (2018). A critical review on speciation, mobilization and toxicity of lead in soil-microbe-plant system and bioremediation strategies. Ecotoxicol. Environ. Saf..

[120] Wuana R.A., Okieimen F.E. (2011). Heavy Metals in Contaminated Soils: A Review of Sources, Chemistry, Risks and Best Available Strategies for Remediation. ISRN Ecol..

[121] Chiou W.Y., Hsu F.C. (2019). Copper toxicity and prediction models of copper content in leafy vegetables. Sustainability.

[122] Bjuhr J. (2007). Trace metals in soils irrigated with waste water in a periurban area downstream Hanoi City, Vietnam. Semin. Pap..

[123] Kopittke P.M., Menzies N.W., de Jonge M.D., Mckenna B.A., Donner E., Webb R.I., Paterson D.J., Howard D.L., Ryan C.G., Glover C.J. (2011). In situ distribution and speciation of toxic copper, nickel, and zinc in hydrated roots of cowpea. Plant Physiol..

[124] Adrees M., Ali S., Rizwan M., Ibrahim M., Abbas F., Farid M., Zia-ur-Rehman M., Irshad M.K., Bharwana S.A. (2015). The effect of excess copper on growth and physiology of important food crops: A review. Environ. Sci. Pollut. Res..

[125] Barbosa R.H., Tabaldi L.A., Miyazaki F.R., Pilecco M., Kassab S.O., Bigaton D. (2013). Absorção foliar de cobre por plantas de milho: Efeitos no crescimento e rendimento. Cienc. Rural.

[126] Aly A.A., Mohamed A.A. (2012). The impact of copper ion on growth, thiol compounds and lipid peroxidation in two maize cultivars (Zea mays L.) grown in vitro. Aust. J. Crop Sci..

[127] Song C., Yan Y., Rosado A., Zhang Z., Castellarin S.D. (2019). ABA alleviates uptake and accumulation of zinc in grapevine (Vitis vinifera l.) by inducing expression of ZIP and detoxification-related genes. Front. Plant Sci..

[128] Broadley M.R., White P.J., Hammond J.P., Zelko I., Lux A. (2007). Zinc in plants: Tansley review. New Phytol..

[129] Hafeez B. (2013). Role of Zinc in Plant Nutrition- A Review. Am. J. Exp. Agric..

[130] Liang J., Yang W. (2019). Effects of Zinc and Copper Stress on Antioxidant System of Olive Leaves. IOP Conf. Ser. Earth Environ. Sci..

[131] Ebbs S.D., Kochian L.V. (1997). Toxicity of Zinc and Copper to Brassica Species: Implications for Phytoremediation. J. Environ. Qual..

[132] Hammerschmitt R.K., Tiecher T.L., Facco D.B., Silva L.O.S., Schwalbert R., Drescher G.L., Trentin E., Somavilla L.M., Kulmann M.S.S., Silva I.C.B. (2020). Copper and zinc distribution and toxicity in ‘Jade’ / ‘Genovesa’ young peach tree. Sci. Hortic..

[133] Balafrej H., Bogusz D., Abidine Triqui Z.E., Guedira A., Bendaou N., Smouni A., Fahr M. (2020). Zinc hyperaccumulation in plants: A review. Plants.

[134] Loi N.N., Sanzharova N.I., Shchagina N.I., Mironova M.P. (2018). The Effect of Cadmium Toxicity on the Development of Lettuce Plants on Contaminated Sod-Podzolic Soil. Russ. Agric. Sci..

[135] Kabata-Pendias A., Kabata-Pendias A. (2010). Trace Elements in Plants. Trace Elements in Soils and Plants Fourth Edition.

[136] Zhou J., Zhang Z., Zhang Y., Wei Y., Jiang Z. (2018). Effects of lead stress on the growth, physiology, and cellular structure of privet seedlings. PLoS ONE.

[137] Panagos P., Ballabio C., Lugato E., Jones A., Borrelli P., Scarpa S., Orgiazzi A., Montanarella L. (2018). Potential Sources of Anthropogenic Copper Inputs to European Agricultural Soils. Sustainability.

[138] Guan Q., Wang F., Xu C., Pan N., Lin J., Zhao R., Yang Y., Luo H. (2018). Source apportionment of heavy metals in agricultural soil based on PMF: A case study in Hexi Corridor, northwest China. Chemosphere.

[139] Plum L.M., Rink L., Haase H. (2010). The Essential Toxin: Impact of Zinc on Human Health. Int. J. Environ. Res. Public Health.

[140] Paoli D., Giannandrea F., Gallo M., Turci R., Cattaruzza M.S., Lombardo F., Lenzi A., Gandini L. (2015). Exposure to polychlorinated biphenyls and hexachlorobenzene, semen quality and testicular cancer risk. J. Endocrinol. Invest..

[141] (2017). World Health Organization (WHO) Agrochemicals, Health and Environment: Directory of Resources. https://www.who.int/heli/risks/toxics/chemicalsdirectory/en/.

[142] El-Wakeil N., Gaafar N., Sallam A., Volkmar C., Trdan S. (2013). Side Effects of Insecticides on Natural Enemies and Possibility of Their Integration in Plant Protection Strategies. Insecticides - Development of Safer and More Effective Technologies.

[143] Zaka S.M., Iqbal N., Saeed Q., Akrem A., Batool M., Khan A.A., Anwar A., Bibi M., Azeem S., Rizvi D.E.N. (2019). Toxic effects of some insecticides, herbicides, and plant essential oils against Tribolium confusum Jacquelin du val (Insecta: Coleoptera: Tenebrionidae). Saudi J. Biol. Sci..

[144] Ligor M., Bukowska M., Ratiu I.-A., Gadzała-Kopciuch R., Buszewski B. (2020). Determination of Neonicotinoids in Honey Samples Originated from Poland and Other World Countries. Molecules.

[145] Sharma A., Kumar V., Thukral A.K., Bhardwaj R. (2019). Responses of plants to pesticide toxicity: An overview. Planta Daninha.

[146] Sharma A., Kumar V., Kumar R., Shahzad B., Thukral A.K., Bhardwaj R., Tejada Moral M. (2018). Brassinosteroid-mediated pesticide detoxification in plants: A mini-review. Cogent Food Agric..

[147] Biondi A., Desneux N., Siscaro G., Zappalà L. (2012). Using organic-certified rather than synthetic pesticides may not be safer for biological control agents: Selectivity and side effects of 14 pesticides on the predator Orius laevigatus. Chemosphere.

[148] Han Y., Mo R., Yuan X., Zhong D., Tang F., Ye C., Liu Y. (2017). Pesticide residues in nut-planted soils of China and their relationship between nut/soil. Chemosphere.

[149] Nannipieri P., Ascher J., Ceccherini M., Landi L., Pietramellara G., Renella G. (2003). Microbial diversity and soil functions. Eur. J. Soil Sci..

[150] Filimon M.N., Voia S.O., Popescu R., Dumitrescu G., Ciochina L.P., Mituletu M., Vlad D.C. (2015). The effect of some insecticides on soil microorganisms based on enzymatic and bacteriological analyses. Rom. Biotechnol. Lett..

[151] AL-Ani M.A.M., Hmoshi R.M., Kanaan I.A., Thanoon A.A. (2019). Effect of pesticides on soil microorganisms. J. Phys. Conf. Ser..

[152] Yousaf S., Khan S., Aslam M.T. (2013). Effect of pesticides on the soil microbial activity. Pak. J. Zool..

[153] Goswami M.R., Pati U.K., Chowdhury A., Mukhopadhyay A. (2013). Studies on the effect of cypermethrin on soil microbial biomass and its activity in an alluvial soil. Int. J. Agric. Food Sci..

[154] Haleem A.M., Kasim S.A., Al-Timimy J.A. (2013). Effect of some organophosphorous insecticides on soil microorganisms populations under lab condition. World Environ..

[155] Madhaiyan M., Poonguzhali S., Hari K., Saravanan V.S., Sa T. (2006). Influence of pesticides on the growth rate and plant-growth promoting traits of Gluconacetobacter diazotrophicus. Pestic. Biochem. Physiol..

[156] Sannino F., Gianfreda L. (2001). Pesticide influence on soil enzymatic activities. Chemosphere.

[157] Pandey S., Singh D.K. (2004). Total bacterial and fungal population after chlorpyrifos and quinalphos treatments in groundnut (Arachis hypogaea L.) soil. Chemosphere.

[158] Niewiadomska A., Sawicka A. (2002). Effect of Carbendazim, Imazetapir and Thiram on Nitrogenase Activity, Number of Microorganisms in Soil and Yield of Hybrid Lucerne (Medicago media). Polish J. Environ. Stud..

[159] Madhuri R.J., Rangaswamy V. (2002). Influence of selected insecticides on phosphatase activity in groundnut (Arachis hypogeae L.) soils. J. Environ. Biol..

[160] Mayanglambam T., Vig K., Singh D.K. (2005). Quinalphos persistence and leaching under field conditions and effects of residues on dehydrogenase and alkaline phosphomonoesterases activities in soil. Bull. Environ. Contam. Toxicol..

[161] Milošević N.A., Govedarica M.M. (2002). Effect of herbicides on microbiological properties of soil. Zb. Matice Srp. za Prir. Nauk..

[162] Kremer R.J., Means N.E. (2009). Glyphosate and glyphosate-resistant crop interactions with rhizosphere microorganisms. Eur. J. Agron..

[163] Chen F., Dixon R.A. (2007). Lignin modification improves fermentable sugar yields for biofuel production. Nat. Biotechnol..

[164] Mishra P.K., Ekielski A. (2019). The self-assembly of lignin and its application in nanoparticle synthesis: A short review. Nanomaterials.

[165] Hussain S., Siddique T., Saleem M., Arshad M., Khalid A. (2009). Impact of pesticides on soil microbial diversity, enzymes, and biochemical reactions. Adv. Agron..

[166] Arif M.A.S., Houwen F., Verstraete W. (1996). Agricultural factors affecting methane oxidation in arable soil. Biol. Fertil. Soils.

[167] Fabra A., Duffard R., De Duffard A.E. (1997). Toxicity of 2, 4-dichlorophenoxyacetic acid to Rhizobium sp in pure culture. Bull. Environ. Contam. Toxicol..

[168] Chalam A.V., Sasikala C., Ramana C.V., Uma N.R., Rao P.R. (1997). Effect of pesticides on the diazotrophic growth and nitrogenase activity of purple nonsulfur bacteria. Bull. Environ. Contam. Toxicol..

[169] Santos A., Flores M. (1995). Effects of glyphosate on nitrogen fixation of free-living heterotrophic bacteria. Lett. Appl. Microbiol..

[170] Guo P., Zhu L., Wang J., Wang J., Xie H., Lv D. (2015). Enzymatic activities and microbial biomass in black soil as affected by azoxystrobin. Environ. Earth Sci..

[171] Chatterjee N.S., Gupta S., Varghese E. (2013). Degradation of metaflumizone in soil: Impact of varying moisture, light, temperature, atmospheric CO2 level, soil type and soil sterilization. Chemosphere.

[172] Wightwick A.M., Reichman S.M., Menzies N.W., Allinson G. (2013). The effects of copper hydroxide, captan and trifloxystrobin fungicides on soil phosphomonoesterase and urease activity. Water Air, Soil Pollut..

[173] Baćmaga M., Kucharski J., Wyszkowska J. (2015). Microbial and enzymatic activity of soil contaminated with azoxystrobin. Environ. Monit. Assess..

[174] Baćmaga M., Wyszkowska J., Kucharski J. (2016). The effect of the Falcon 460 EC fungicide on soil microbial communities, enzyme activities and plant growth. Ecotoxicology.

[175] Adams G.O., Fufeyin P.T., Okoro S.E., Ehinomen I. (2015). Bioremediation, biostimulation and bioaugmention: A review. Int. J. Environ. Bioremediation Biodegrad..

[176] Baćmaga M., Wyszkowska J., Kucharski J. (2018). The influence of chlorothalonil on the activity of soil microorganisms and enzymes. Ecotoxicology.

[177] Saha A., Pipariya A., Bhaduri D. (2016). Enzymatic activities and microbial biomass in peanut field soil as affected by the foliar application of tebuconazole. Environ. Earth Sci..

[178] Sharma A., Kumar V., Kohli S.K., Thukral A.K., Bhardwaj R. (2015). Phytochemicals in Brassica juncea L. seedlings under imidacloprid-epibrassinolide treatment using GC-MS. J. Chem. Pharm. Res..

[179] Parween T., Jan S., Fatma T. (2011). Alteration in nitrogen metabolism and plant growth during different developmental stages of green gram (Vigna radiata L.) in response to chlorpyrifos. Acta Physiol. Plant..

[180] Parween T., Jan S., Fatma T. (2012). Evaluation of oxidative stress in Vigna radiata L. in response to chlorpyrifos. Int. J. Environ. Sci. Technol..

[181] Sharma A., Kumar V., Singh R., Thukral A.K., Bhardwaj R. (2015). 24-Epibrassinolide induces the synthesis of phytochemicals effected by imidacloprid pesticide stress in Brassica juncea L. J. Pharmacogn. Phytochem..

[182] Boutin C., Strandberg B., Carpenter D., Mathiassen S.K., Thomas P.J. (2014). Herbicide impact on non-target plant reproduction: What are the toxicological and ecological implications?. Environ. Pollut..

[183] Kaya A., Yigit E. (2014). The physiological and biochemical effects of salicylic acid on sunflowers (Helianthus annuus) exposed to flurochloridone. Ecotoxicol. Environ. Saf..

[184] Kaya A., Doganlar Z.B. (2016). Exogenous jasmonic acid induces stress tolerance in tobacco (Nicotiana tabacum) exposed to imazapic. Ecotoxicol. Environ. Saf..

[185] Fernandes B., Soares C., Braga C., Rebotim A., Ferreira R., Ferreira J., Fidalgo F., Pereira R., Cachada A. (2020). Ecotoxicological Assessment of a Glyphosate-Based Herbicide in Cover Plants: Medicago sativa L. as a Model Species. Appl. Sci..

[186] Chen S., Yang L., Hu M., Liu J. (2011). Biodegradation of fenvalerate and 3-phenoxybenzoic acid by a novel Stenotrophomonas sp. strain ZS-S-01 and its use in bioremediation of contaminated soils. Appl. Microbiol. Biotechnol..

[187] Jezierska-Tys S., Rutkowska A. (2013). Soil response to chemicals used in a field experiment. Int. Agrophys..

[188] Zhou Y., Xia X., Yu G., Wang J., Wu J., Wang M., Yang Y., Shi K., Yu Y., Chen Z. (2015). Brassinosteroids play a critical role in the regulation of pesticide metabolism in crop plants. Sci. Rep..

[189] Xia X.J., Huang Y.Y., Wang L., Huang L.F., Yu Y.L., Zhou Y.H., Yu J.Q. (2006). Pesticides-induced depression of photosynthesis was alleviated by 24-epibrassinolide pretreatment in Cucumis sativus L. Pestic. Biochem. Physiol..

[190] Ijaz M., Mahmood K., Honermeier B. (2015). Interactive role of fungicides and plant growth regulator (Trinexapac) on seed yield and oil quality of winter rapeseed. Agronomy.

[191] Shahid M., Ahmed B., Zaidi A., Khan M.S. (2018). Toxicity of fungicides to Pisum sativum: A study of oxidative damage, growth suppression, cellular death and morpho-anatomical changes. RSC Adv..

[192] Ahemad M., Khan M.S. (2011). Assessment of plant growth promoting activities of rhizobacterium Pseudomonas putida under insecticide-stress. Microbiol. J..

[193] Ahemad M., Khan M.S. (2010). Comparative toxicity of selected insecticides to pea plants and growth promotion in response to insecticide-tolerant and plant growth promoting Rhizobium leguminosarum. Crop Prot..

[194] Saladin G., Clément C., Livingston J.V. (2005). Physiological Side Effects of Pesticides on Non-target Plants. Agriculture and Soil Pollution: New Research.

[195] Petit A.N., Fontaine F., Vatsa P., Clément C., Vaillant-Gaveau N. (2012). Fungicide impacts on photosynthesis in crop plants. Photosynth. Res..

[196] Sáez F., Pozo C., Gómez M.A., Martínez-Toledo M.V., Rodelas B., Gónzalez-López J. (2006). Growth and denitrifying activity of Xanthobacter autotrophicus CECT 7064 in the presence of selected pesticides. Appl. Microbiol. Biotechnol..

[197] Kanissery R., Gairhe B., Kadyampakeni D., Batuman O., Alferez F. (2019). Glyphosate: Its Environmental Persistence and Impact on Crop Health and Nutrition. Plants.

[198] Uwizeyimana H., Wang M., Chen W., Khan K. (2017). The eco-toxic effects of pesticide and heavy metal mixtures towards earthworms in soil. Environ. Toxicol. Pharmacol..

[199] Chen C., Wang Y., Qian Y., Zhao X., Wang Q. (2015). The synergistic toxicity of the multiple chemical mixtures: Implications for risk assessment in the terrestrial environment. Environ. Int..

[200] Wang Y., Chen C., Qian Y., Zhao X., Wang Q. (2015). Ternary toxicological interactions of insecticides, herbicides, and a heavy metal on the earthworm Eisenia fetida. J. Hazard. Mater..

[201] Chen Y.X., Lin Q., He Y.F., Tian G.M. (2004). Behavior of Cu and Zn under combined pollution of 2,4-dichlorophenol in the planted soil. Plant Soil.

[202] Chao L., Zhou Q.X., Chen S., Cui S., Wang M.E. (2007). Single and joint stress of acetochlor and Pb on three agricultural crops in northeast China. J. Environ. Sci..

[203] Divisekara T., Navaratne A.N., Abeysekara A.S.K. (2018). Impact of a commercial glyphosate formulation on adsorption of Cd(II) and Pb(II) ions on paddy soil. Chemosphere.

[204] Liang J., Zhou Q. (2003). Single and Binary-Combined Toxicity of Methamidophos, Acetochlor and Copper Acting on Earthworms Esisenia Foelide. Bull. Environ. Contam. Toxicol..

[205] Liu J., Xie J., Chu Y., Sun C., Chen C., Wang Q. (2008). Combined effect of cypermethrin and copper on catalase activity in soil. J. Soils Sediments.

[206] García-Gómez C., Babín M., García S., Almendros P., Pérez R.A., Fernández M.D. (2019). Joint effects of zinc oxide nanoparticles and chlorpyrifos on the reproduction and cellular stress responses of the earthworm Eisenia andrei. Sci. Total Environ..

[207] Huang H., Xiong Z.T. (2009). Toxic effects of cadmium, acetochlor and bensulfuron-methyl on nitrogen metabolism and plant growth in rice seedlings. Pestic. Biochem. Physiol..

[208] Liu N., Zhong G., Zhou J., Liu Y., Pang Y., Cai H., Wu Z. (2019). Separate and combined effects of glyphosate and copper on growth and antioxidative enzymes in Salvinia natans (L.) All. Sci. Total Environ..

[209] Gill S.S., Tuteja N. (2010). Reactive oxygen species and antioxidant machinery in abiotic stress tolerance in crop plants. Plant Physiol. Biochem..

[210] Abouziena H.F., Elmergawi R.A., Sharma S., Omar A.A., Singh M. (2009). Zinc Antagonizes Glyphosate Efficacy on Yellow Nutsedge ( Cyperus esculentus ). Weed Sci..

[211] Khlifi R., Hamza-Chaffai A. (2010). Head and neck cancer due to heavy metal exposure via tobacco smoking and professional exposure: A review. Toxicol. Appl. Pharmacol..

[212] Sankhla M.S., Kumar R. (2019). Contaminant of Heavy Metals in Groundwater & its Toxic Effects on Human Health & Environment. Int. J. Environ. Sci. Nat. Resour..

[213] Jiang X., Zou B., Feng H., Tang J., Tu Y., Zhao X. (2019). Spatial distribution mapping of Hg contamination in subclass agricultural soils using GIS enhanced multiple linear regression. J. Geochemical Explor..

[214] Xiao R., Guo D., Ali A., Mi S., Liu T., Ren C., Li R., Zhang Z. (2019). Accumulation, ecological-health risks assessment, and source apportionment of heavy metals in paddy soils: A case study in Hanzhong, Shaanxi, China. Environ. Pollut..

[215] Lamas G.A., Navas-Acien A., Mark D.B., Lee K.L. (2016). Heavy Metals, Cardiovascular Disease, and the Unexpected Benefits of Chelation Therapy. J. Am. Coll. Cardiol..

[216] Ma Y., Egodawatta P., McGree J., Liu A., Goonetilleke A. (2016). Human health risk assessment of heavy metals in urban stormwater. Sci. Total Environ..

[217] Wang F., Guan Q., Tian J., Lin J., Yang Y., Yang L., Pan N. (2020). Contamination characteristics, source apportionment, and health risk assessment of heavy metals in agricultural soil in the Hexi Corridor. CATENA.

[218] Yang F., Massey I.Y. (2019). Exposure routes and health effects of heavy metals on children. Biometals.

[219] Chunhabundit R. (2016). Cadmium exposure and potential health risk from foods in contaminated area, Thailand. Toxicol. Res..

[220] Schoeters G., HOND E.D.E.N., Zuurbier M., Naginiene R., Van den Hazel P., Stilianakis N., Ronchetti R., Koppe J.G. (2006). Cadmium and children: Exposure and health effects. Acta Paediatr..

[221] Sherief L.M., Abdelkhalek E.R., Gharieb A.F., Sherbiny H.S., Usef D.M., Almalky M.A.A., Kamal N.M., Salama M.A., Gohar W. (2015). Cadmium status among pediatric cancer patients in Egypt. Medicine (Baltimore).

[222] Gardner R.M., Kippler M., Tofail F., Bottai M., Hamadani J., Grandér M., Nermell B., Palm B., Rasmussen K.M., Vahter M. (2013). Environmental exposure to metals and children’s growth to age 5 years: A prospective cohort study. Am. J. Epidemiol..

[223] Maret W., Sigel A., Sigel H., Sigel R.K.O. (2017). The Bioinorganic Chemistry of Lead in the Context of Its Toxicity. Lead: Its Effects on Environment and Health.

[224] McMichael J.R., Stoff B.K. (2018). Surma eye cosmetic in Afghanistan: A potential source of lead toxicity in children. Eur. J. Pediatr..

[225] Evens A., Hryhorczuk D., Lanphear B.P., Rankin K.M., Lewis D.A., Forst L., Rosenberg D. (2015). The impact of low-level lead toxicity on school performance among children in the Chicago Public Schools: A population-based retrospective cohort study. Environ. Health.

[226] Pfadenhauer L.M., Burns J., Rohwer A., Rehfuess E.A. (2014). A protocol for a systematic review of the effectiveness of interventions to reduce exposure to lead through consumer products and drinking water. Syst. Rev..

[227] Johnson W.T., Lieberman H.R., Kanarek R.B., Prasad C. (2005). Nutritional neuroscience. Nutritional Neuroscience.

[228] Zhou G., Ji X., Cui N., Cao S., Liu C., Liu J. (2015). Association between serum copper status and working memory in schoolchildren. Nutrients.

[229] Roberts E.A., Socha P., Członkowska A., Schilsky M.L. (2017). Wilson disease in children. Handbook of clinical neurology.

[230] Arsenault J.E., Brown K.H. (2003). Zinc intake of US preschool children exceeds new dietary reference intakes. Am. J. Clin. Nutr..

[231] Black J.L., Piñero D.J., Parekh N. (2009). Zinc and cognitive development in children: Perspectives from international studies. Top. Clin. Nutr..

[232] Shaikhkhalil A.K., Curtiss J., Puthoff T.D., Valentine C.J. (2014). Enteral zinc supplementation and growth in extremely-low-birth-weight infants with chronic lung disease. J. Pediatr. Gastroenterol. Nutr..

[233] Jeejeebhoy K. (2009). Zinc: An essential trace element for parenteral nutrition. Gastroenterology.

[234] Lim K.H.C., Riddell L.J., Nowson C.A., Booth A.O., Szymlek-Gay E.A. (2013). Iron and zinc nutrition in the economically-developed world: A review. Nutrients.

[235] Chandra R.K. (1984). Excessive intake of zinc impairs immune responses. JAMA.

[236] Willoughby J.L., Bowen C.N. (2014). Zinc deficiency and toxicity in pediatric practice. Curr. Opin. Pediatr..

[237] Avenant-Oldewage A., Marx H.M. (2000). Bioaccumulation of chromium, copper and iron in the organs and tissues of Clarias gariepinus in the Olifants River, Kruger National Park. Water SA.

[238] Jiang L.-F., Yao T.-M., Zhu Z.-L., Wang C., Ji L.-N. (2007). Impacts of Cd (II) on the conformation and self-aggregation of Alzheimer’s tau fragment corresponding to the third repeat of microtubule-binding domain. Biochim. Biophys. Acta-Proteins Proteomics.

[239] Reyes-Hinojosa D., Lozada-Pérez C.A., Cuevas Y.Z., López-Reyes A., Martínez-Nava G., Fernández-Torres J., Olivos-Meza A., Landa-Solis C., Gutiérrez-Ruiz M.C., Del Castillo E.R. (2019). Toxicity of cadmium in musculoskeletal diseases. Environ. Toxicol. Pharmacol..

[240] Kumar S., Sharma A. (2019). Cadmium toxicity: Effects on human reproduction and fertility. Rev. Environ. Health.

[241] Esteban-Vasallo M.D., Aragonés N., Pollan M., López-Abente G., Perez-Gomez B. (2012). Mercury, cadmium, and lead levels in human placenta: A systematic review. Environ. Health Perspect..

[242] Fatima G., Raza A.M., Hadi N., Nigam N., Mahdi A.A. (2019). Cadmium in human diseases: It’s more than just a mere metal. Indian J. Clin. Biochem..

[243] Wani A.L., Ara A., Usmani J.A. (2015). Lead toxicity: A review. Interdiscip. Toxicol..

[244] Gundacker C., Hengstschläger M. (2012). The role of the placenta in fetal exposure to heavy metals. Wien. Med. Wochenschr..

[245] Malik R.N., Zeb N. (2009). Assessment of environmental contamination using feathers of Bubulcus ibis L., as a biomonitor of heavy metal pollution, Pakistan. Ecotoxicology.

[246] Goyer R.A., Clarkson T.W., Klaassen C.D. (2001). Toxic effects of metals. Casarett and Doull’s Toxicology: The Basic Science of Poisons.

[247] Ogwuegbu M.O., Ijioma M.A. Effects of certain heavy metals on the population due to mineral exploitation. Proceedings of the International Conference on Scientific and Environmental Issues in the Population, Environment and Sustainable Development in Nigeria.

[248] Uriu-Adams J.Y., Keen C.L. (2005). Copper, oxidative stress, and human health. Mol. Asp. Med..

[249] Gamakaranage C.S.S.K., Rodrigo C., Weerasinghe S., Gnanathasan A., Puvanaraj V., Fernando H. (2011). Complications and management of acute copper sulphate poisoning; a case discussion. J. Occup. Med. Toxicol..

[250] Hordyjewska A., Popiołek Ł., Kocot J. (2014). The many “faces” of copper in medicine and treatment. BioMetals.

[251] Harris E.D. (2000). Cellular copper transport and metabolism. Annu. Rev. Nutr..

[252] Oe S., Miyagawa K., Honma Y., Harada M. (2016). Copper induces hepatocyte injury due to the endoplasmic reticulum stress in cultured cells and patients with Wilson disease. Exp. Cell Res..

[253] Hambidge K.M., Mertz W. (1986). Zinc. Trace Elements in Human and Animal Nutrition.

[254] Morris D.R., Levenson C.W., Aschner M.C.L. (2017). Neurotoxicity of Zinc. Neurotoxicity of Metals.

[255] Bush A.I. (2013). The metal theory of Alzheimer’s disease. J. Alzheimer’s Dis..

[256] Planchart A., Green A., Hoyo C., Mattingly C.J. (2018). Heavy metal exposure and metabolic syndrome: Evidence from human and model system studies. Curr. Environ. Health Rep..

[257] Sabarwal A., Kumar K., Singh R.P. (2018). Hazardous effects of chemical pesticides on human health–Cancer and other associated disorders. Environ. Toxicol. Pharmacol..

[258] Luo D., Zhou T., Tao Y., Feng Y., Shen X., Mei S. (2016). Exposure to organochlorine pesticides and non-Hodgkin lymphoma: A meta-analysis of observational studies. Sci. Rep..

[259] Anderson S.E., Meade B.J. (2014). Potential Health Effects Associated with Dermal Exposure to Occupational Chemicals. Environ. Health Insights.

[260] Fareed M., Kesavachandran C.N., Pathak M.K., Bihari V., Kuddus M., Srivastava A.K. (2012). Visual disturbances with cholinesterase depletion due to exposure of agricultural pesticides among farm workers. Toxicol. Environ. Chem..

[261] Amaral A.F.S. (2014). Pesticides and Asthma: Challenges for Epidemiology. Front. Public Health.

[262] Nie H., Jacobi H.F., Strach K., Xu C., Zhou H., Liebetrau J. (2015). Mono-fermentation of chicken manure: Ammonia inhibition and recirculation of the digestate. Bioresour. Technol..

[263] Bonner M.R., Freeman L.E.B., Hoppin J.A., Koutros S., Sandler D.P., Lynch C.F., Hines C.J., Thomas K., Blair A., Alavanja M.C.R. (2017). Occupational Exposure to Pesticides and the Incidence of Lung Cancer in the Agricultural Health Study. Environ. Health Perspect..

[264] Polanco Rodríguez Á.G., Riba López M.I., DelValls Casillas T.Á., Araujo León J.A., Mahjoub O., Prusty A.K. (2017). Monitoring of organochlorine pesticides in blood of women with uterine cervix cancer. Environ. Pollut..

[265] Hernández A.F., Parrón T., Alarcón R. (2011). Pesticides and asthma. Curr. Opin. Allergy Clin. Immunol..

[266] Azandjeme C., Bouchard M., Fayomi B., Djrolo F., Houinato D., Delisle H. (2013). Growing Burden of Diabetes in Sub- Saharan Africa: Contribution of Pesticides?. Curr. Diabetes Rev..

[267] Freire C., Koifman S. (2012). NeuroToxicology Pesticide exposure and Parkinson ’ s disease: Epidemiological evidence of association. Neurotoxicology.

[268] Brouwer M., Huss A., van der Mark M., Nijssen P.C.G., Mulleners W.M., Sas A.M.G., van Laar T., de Snoo G.R., Kromhout H., Vermeulen R.C.H. (2017). Environmental exposure to pesticides and the risk of Parkinson’s disease in the Netherlands. Environ. Int..

[269] Frazier L.M. (2007). Reproductive Disorders Associated with Pesticide Exposure. J. Agromedicine.

[270] Mehrpour O., Karrari P., Zamani N., Tsatsakis A.M., Abdollahi M. (2014). Occupational exposure to pesticides and consequences on male semen and fertility: A review. Toxicol. Lett..

[271] Kabir E.R., Rahman M.S., Rahman I. (2015). A review on endocrine disruptors and their possible impacts on human health. Environ. Toxicol. Pharmacol..

[272] Garry V.F. (2004). Pesticides and children. Toxicol. Appl. Pharmacol..

[273] Grover P., Danadevi K., Mahboob M., Rozati R., Banu B.S., Rahman M.F. (2003). Evaluation of genetic damage in workers employed in pesticide production utilizing the Comet assay. Mutagenesis.

[274] Peluso M., Merlo F., Munnia A., Bolognesi C., Puntoni R., Parodi S. (1996). 32P-postlabeling detection of DNA adducts in peripheral white blood cells of greenhouse floriculturists from Western Liguria, Italy. Cancer Epidemiol. Biomarkers Prev..

[275] Edwards T.M., Myers J.P. (2007). Environmental Exposures and Gene Regulation in Disease Etiology. Environ. Health Perspect..

[276] Carbonell E., Xamena N., Creus A., Marcos R. (1993). Cytogenetic biomonitoring in a Spanish group of agricultural workers exposed to pesticides. Mutagenesis.

[277] Dong L.M., Potter J.D., White E., Ulrich C.M., Cardon L.R., Peters U. (2008). Genetic Susceptibility to Cancer: The role of polymorphisms in candidate genes. JAMA.

[278] Wallace D.R., Buha Djordjevic A. (2020). Heavy metal and pesticide exposure: A mixture of potential toxicity and carcinogenicity. Curr. Opin. Toxicol..

[279] Adamkovicova M., Toman R., Cabaj M., Massanyi P., Martiniakova M., Omelka R., Krajcovicova V., Duranova H. (2014). Effects of subchronic exposure to cadmium and diazinon on testis and epididymis in rats. Sci. World J..

[280] He W., Guo W., Qian Y., Zhang S., Ren D., Liu S. (2015). Synergistic hepatotoxicity by cadmium and chlorpyrifos: Disordered hepatic lipid homeostasis. Mol. Med. Rep..

[281] Seo S., Choi S., Kim K., Kim S.M., Park S.M. (2019). Association between urban green space and the risk of cardiovascular disease: A longitudinal study in seven Korean metropolitan areas. Environ. Int..

